# *Tex19*.*1* promotes *Spo11*-dependent meiotic recombination in mouse spermatocytes

**DOI:** 10.1371/journal.pgen.1006904

**Published:** 2017-07-14

**Authors:** James H. Crichton, Christopher J. Playfoot, Marie MacLennan, David Read, Howard J. Cooke, Ian R. Adams

**Affiliations:** MRC Human Genetics Unit, MRC Institute of Genetics and Molecular Medicine, University of Edinburgh, Western General Hospital, Edinburgh, United Kingdom; The University of Texas MD Anderson Cancer Center, UNITED STATES

## Abstract

Meiosis relies on the SPO11 endonuclease to generate the recombinogenic DNA double strand breaks (DSBs) required for homologous chromosome synapsis and segregation. The number of meiotic DSBs needs to be sufficient to allow chromosomes to search for and find their homologs, but not excessive to the point of causing genome instability. Here we report that the mammal-specific gene *Tex19*.*1* promotes *Spo11*-dependent recombination in mouse spermatocytes. We show that the chromosome asynapsis previously reported in *Tex19*.*1*^*-/-*^ spermatocytes is preceded by reduced numbers of recombination foci in leptotene and zygotene. *Tex19*.*1* is required for normal levels of early *Spo11*-dependent recombination foci during leptotene, but not for upstream events such as MEI4 foci formation or accumulation of H3K4me3 at recombination hotspots. Furthermore, we show that mice carrying mutations in *Ubr2*, which encodes an E3 ubiquitin ligase that interacts with TEX19.1, phenocopy the *Tex19*.*1*^*-/-*^ recombination defects. These data suggest that *Tex19*.*1* and *Ubr2* are required for mouse spermatocytes to accumulate sufficient *Spo11*-dependent recombination to ensure that the homology search is consistently successful, and reveal a hitherto unknown genetic pathway promoting meiotic recombination in mammals.

## Author summary

Meiosis is a specialised type of cell division that occurs during sperm and egg development to reduce chromosome number prior to fertilisation. Recombination is a key step in meiosis as it facilitates the pairing of homologous chromosomes prior to their reductional division, and generates new combinations of genetic alleles for transmission in the next generation. Regulating the amount of recombination is key for successful meiosis: as this process involves making many simultaneous breaks in the DNA, too much will likely cause mutations, chromosomal re-arrangements and genetic instability; whereas too little causes defects in homologous chromosome pairing prior to the meiotic divisions. This study identifies a genetic pathway required to generate robust meiotic recombination in mouse spermatocytes. We show that male mice with mutations in *Tex19*.*1* or *Ubr2*, which encodes an E3 ubiquitin ligase that interacts with TEX19.1, do not generate sufficient meiotic recombination. We show that the defects in these mutants impact on recombination early in meiosis when programmed DNA double strand breaks are being made and processed. This defect likely contributes to the chromosome synapsis and meiotic progression phenotypes previously described in these mutant mice. This study has implications for our understanding of how this fundamental aspect of genetics and inheritance is controlled.

## Introduction

Recombination plays key roles in meiosis and gametogenesis through facilitating the pairing and reductional segregation of homologous chromosomes, and by increasing genetic variation in the next generation. Meiotic recombination is initiated when programmed DNA double strand breaks (DSBs) are generated during the leptotene stage of the first meiotic prophase. Meiotic DSBs recruit a series of recombination proteins visualised cytologically as recombination foci, and initiate a search for homologous chromosomes thereby promoting homologous chromosome synapsis during zygotene. Recombination foci continue to mature while the chromosomes are fully synapsed in pachytene, and eventually resolve into crossover or non-crossover events. Crossovers exchange large tracts of genetic information between parental chromosomes, increasing genetic diversity in the population. Furthermore, these crossovers, which physically manifest as chiasmata, hold homologs together after they desynapse in diplotene and help to ensure that homologous chromosomes undergo an ordered reductional segregation at anaphase I [1,2].

Meiotic DSBs have a non-random distribution across the genome, and their frequency and location play an important role in shaping the recombination landscape [2,3]. In male mice, a few hundred meiotic DSBs are generated during leptotene, around 20–25 of which mature into crossovers. The positions of meiotic DSBs across the genome are determined by PRDM9, a histone methyltransferase that mediates trimethylation of histone H3 lysine 4 (H3K4me3) at recombination hotspots [4,5]. Meiotic DSBs are generated by an endonuclease that comprises SPO11 and TOPOVIBL subunits [2,3,6]. In mice, mutations in *Spo11* result in fewer DSBs during leptotene and zygotene, and defects in the pairing and synapsis of homologous chromosomes [7–9]. The overall amount of SPO11 activity appears to be dynamically controlled at multiple levels during meiotic prophase. At the RNA level, *Spo11* is alternatively spliced into two major isoforms whose relative abundance changes as meiotic prophase proceeds [10–12]. There also appears to be regulation of SPO11 activity at the protein level: negative feedback mechanisms acting through the DNA damage-associated protein kinase ATM prevent excessive *Spo11*-dependent DSBs from being generated during meiosis, potentially limiting any genetic instability caused by errors arising during repair of the DSBs and meiotic arrest caused by unrepaired DSBs [13]; and chromosome synapsis feeds back to locally inhibit SPO11 activity in chromosomal regions that have already synapsed during zygotene [14].

Mutations in genes involved in regulating early stages in meiotic recombination in mammals might be expected to phenocopy *Spo11*^*-/-*^ mutants to some extent in having reduced numbers of DSBs in leptotene, and arrest at pachytene with chromosome asynapsis. One group of genes that is required for chromosome synapsis in mouse spermatocytes, but whose mechanistic role in meiosis is poorly defined, is the germline genome defence genes [15]. These genes are involved in suppressing the activity of retrotransposons in developing germ cells, and mutations in many of them cause defects in progression through the pachytene stage of meiosis [15]. Mutations in one of these germline genome defence genes, *Mael*, which encodes a conserved component of the piRNA pathway, causes de-repression of retrotransposons and a considerable increase in *Spo11*-independent DNA damage [16]. The *Spo11*-independent DNA damage generated in these mutants could potentially reflect the activity of the retrotransposon-encoded endonucleases that generate nicks or breaks in the host DNA to mediate mobilisation of these genetic elements [16]. In contrast, spermatocytes carrying mutations in the DNA methyltransferase accessory factor *Dnmt3l* also de-repress retrotransposons, but have relatively normal levels of DSBs that are aberrantly distributed across the genome [17–19].

The germline specificity in expression of at least a subset of the germline genome-defence genes is achieved through tissue-specific promoter DNA methylation [20]. One of the most methylation sensitive of these genes is *Tex19*.*1* [20,21]. *Tex19*.*1* was originally identified in a screen for testis-specific genes [22], and is one of two rodent paralogs of this mammal-specific gene family [23]. Although TEX19.1 was described as being a nuclear factor with potential roles in maintenance of stem cells or pluripotency [23], subsequent functional studies demonstrated that TEX19.1 is predominantly cytoplasmic in the mouse germline, where it has roles in meiosis and repression of retrotransposons [24,25]. TEX19.1 physically interacts with UBR2 [25], inhibiting the activity of this E3 ubiquitin ligase towards its normal cellular substrates in the N-end rule pathway [26] and promoting its activity towards retrotransposon-encoded proteins [27]. *Tex19*.*1* mutant spermatocytes progress into the pachytene stage of meiotic prophase but frequently contain asynapsed chromosomes and accumulate retrotransposon RNA [24,25]. Thus, *Tex19*.*1* mutants arrest at a similar stage of meiosis as *Dnmt3L* and *Mael* mutants, despite expressing different retrotransposon RNAs [16,17,24]. However, meiotic chromosome synapsis requires multiple upstream events to be executed correctly, and it is not clear if the meiotic defects in *Tex19*.*1* mutant spermatocytes are similar to the defects present in *Dnmt3L* and *Mael* mutant spermatocytes, or if they arise through different mechanisms.

In this study we elucidate why loss of the germline genome defence gene *Tex19*.*1* results in chromosome asynapsis in male meiosis. We show that loss of *Tex19*.*1* generates a meiotic phenotype distinct from either *Mael*^*-/-*^ or *Dnmt3l*^*-/-*^ mutants. Rather loss of *Tex19*.*1* phenocopies hypomorphic *Spo11* mutants and impairs *Spo11*-dependent recombination during the leptotene stage of meiotic prophase. Furthermore, we show that mice lacking the TEX19.1-interacting protein UBR2 phenocopy the recombination defects seen in leptotene *Tex19*.*1*^*-/-*^ spermatocytes. These data show that *Tex19*.*1* and *Ubr2* are required for sufficient SPO11-dependent recombination to ensure robust identification and synapsis of homologous chromosomes in meiotic spermatocytes.

## Results

### Chromosome asynapsis in *Tex19*.*1*^*-/-*^ spermatocytes is not caused by primary defects in synaptonemal complex assembly

*Tex19*.*1* is a DNA methylation-sensitive germline genome defence gene whose expression is primarily restricted to germ cells and pluripotent cells in the embryo [20,22–24]. We and others have previously reported that *Tex19*.*1*^*-/-*^ males have defects in spermatogenesis on a mixed genetic background, and that around 50% of pachytene spermatocytes in *Tex19*.*1*^*-/-*^ testes have asynapsed chromosomes, but the molecular explanation for this defect remains unknown [15,24,25]. Synapsis requires the accurate and timely execution of a number of events in the preceding stages of the first meiotic prophase, including the generation of meiotic DNA double-strand breaks (DSBs) in leptotene, followed by homolog pairing and assembly of the synaptonemal complex (SC) in zygotene [1]. To investigate the molecular basis for the chromosome asynapsis in pachytene *Tex19*.*1*^*-/-*^ spermatocytes we sought to test whether each of these events occurs normally in the absence of *Tex19*.*1*.

**Fig 1:**
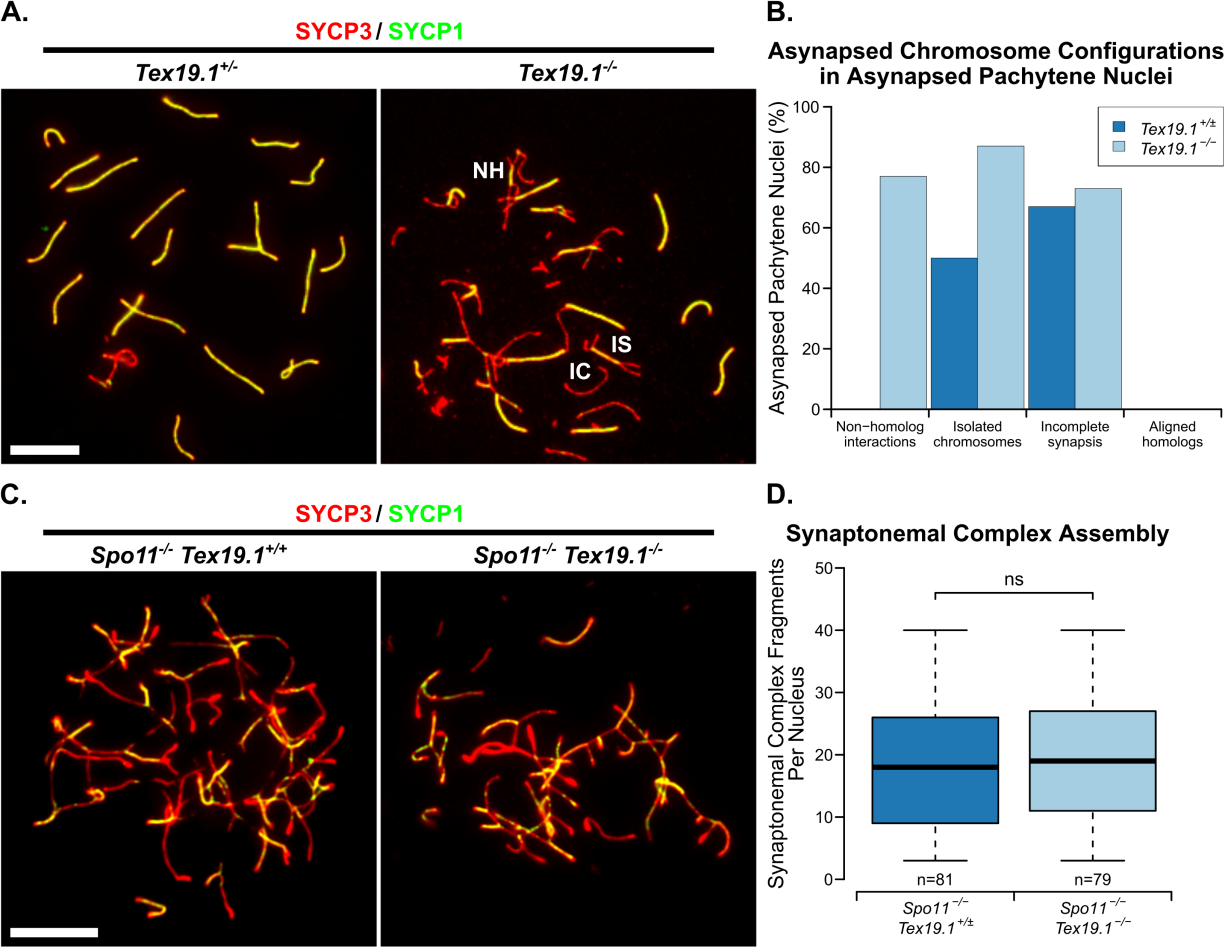
**Asynapsis in *Tex19*.*1*^*-/-*^ Spermatocytes is not Directly Caused by Impaired Synaptonemal Complex Assembly.** (A) Immunostaining of chromosome spreads from *Tex19*.*1*^*+/±*^ and *Tex19*.*1*^*-/-*^ spermatocytes for synaptonemal complex (SC) components SYCP3 (red) and SYCP1 (green). Asynapsed chromosomes assemble SYCP3 but not SYCP1, and examples involving non-homologous interactions (NH), incomplete synapsis between homologs (IS), and isolated chromosomes (IC) are labelled. Non-homologous interactions were identified due to interacting chromosome axes being different lengths, interactions between multiple axes, and/or interactions between different positions along the axes involved. Scale bar 10 μm. (B) Percentage of asynapsed pachytene *Tex19*.*1*^*+/±*^ and *Tex19*.*1*^*-/-*^ spermatocytes exhibiting the indicated categories of asynapsed chromosomes (60 asynapsed pachytene nuclei from 3 *Tex19*.*1*^*-/-*^ mice and 6 asynapsed pachytene nuclei from 3 *Tex19*.*1*^*+/±*^ mice were scored). Fully autosomally synapsed pachytene *Tex19*.*1*^*-/-*^ nuclei were not included in this analysis, and only asynapsed pachytene nuclei containing clearly distinguishable asynapsed chromosome configurations were scored. Each scored asynapsed pachytene nucleus is typically represented in more than one category (C) Immunostaining of chromosome spreads from zygotene-like *Spo11*^*-/-*^ *Tex19*.*1*^*+/±*^ and *Spo11*^*-/-*^ *Tex19*.*1*^*-/-*^ spermatocytes for the SC components SYCP3 (red) and SYCP1 (green). Linear fragments of fully assembled SC can be seen where SYCP3 and SYCP1 co-localise. Scale bar 10 μm. (D) Boxplots showing quantification of SC linear fragments in zygotene-like *Spo11*^*-/-*^ *Tex19*.*1*^*+/±*^ and *Spo11*^*-/-*^ *Tex19*.*1*^*-/-*^ spermatocyte nuclei (17.8±1.1 and 19.2±1.1 linear fragments respectively). n = 79, 79 spreads from 3 mice per genotype. ns indicates no significant difference (Mann-Whitney U test).

First, we confirmed that the meiotic chromosome asynapsis phenotype persists in *Tex19*.*1*^*-/-*^ spermatocytes after backcrossing onto an inbred C57BL/6 genetic background: 65.4% ± 1.3 of *Tex19*.*1*^*-/-*^ pachytene spermatocytes from three animals were asynapsed in this genetic background, significantly higher than the 3.7% ± 1.3 of *Tex19*.*1*^*+/±-*^ control pachytene spermatocytes that were asynapsed (Student’s t-test, p<0.001) (Fig 1A). This is similar to the asynapsis in 50% of *Tex19*.*1*^*-/-*^ pachytene nuclei previously reported for a mixed genetic background [24,25]. To assess whether the chromosome asynapsis in *Tex19*.*1*^*-/-*^ spermatocytes represents defects in SC assembly rather than pairing of homologous chromosomes, we scored the configuration of the asynapsed chromosomes in these asynapsed pachytene *Tex19*.*1*^*-/-*^ nuclei. Defects in assembly of the SC transverse filaments results in asynapsed chromosomes that are aligned in their homolog pairs whereas defective recombination or pairing between homologous chromosomes manifests as isolated asynapsed single chromosomes, partial synapsis between non-homologous chromosomes, and incomplete synapsis between homologous chromosomes [7,8,28,29]. Asynapsed chromosomes in *Tex19*.*1*^*-/-*^ spermatocytes are present in multiple configurations consistent with defects in recombination or homolog pairing, but do not present as asynapsed aligned homolog pairs (Fig 1A, Fig 1B).

To confirm that the asynapsis phenotype in *Tex19*.*1*^*-/-*^ spermatocytes does not represent a primary defect in SC assembly, we quantified the effect of *Tex19*.*1* on the number of SC fragments assembled independently of recombination in a *Spo11*^*-/-*^ genetic background [30]. *Spo11*^*-/-*^ spermatocytes arrest with a zygotene-like SC configuration with complete axial element formation but limited synapsis [7,8]. *Spo11*^*-/-*^ *Tex19*.*1*^*+/±*^ and *Spo11*^*-/-*^ *Tex19*.*1*^*-/-*^ spermatocytes are able to assemble similar amounts of SC in this assay (Fig 1C, Fig 1D), suggesting that loss of *Tex19*.*1* does not severely impair recombination-independent SC assembly. Taken together, these data suggest that the chromosome asynapsis in *Tex19*.*1*^*-/-*^ spermatocytes is likely primarily caused by defects in meiotic recombination and/or homolog pairing rather than a direct defect in SC assembly.

### Chromosome asynapsis in *Tex19*.*1*^*-/-*^ spermatocytes is associated with an earlier reduction in the number of meiotic recombination foci

**Fig 2:**
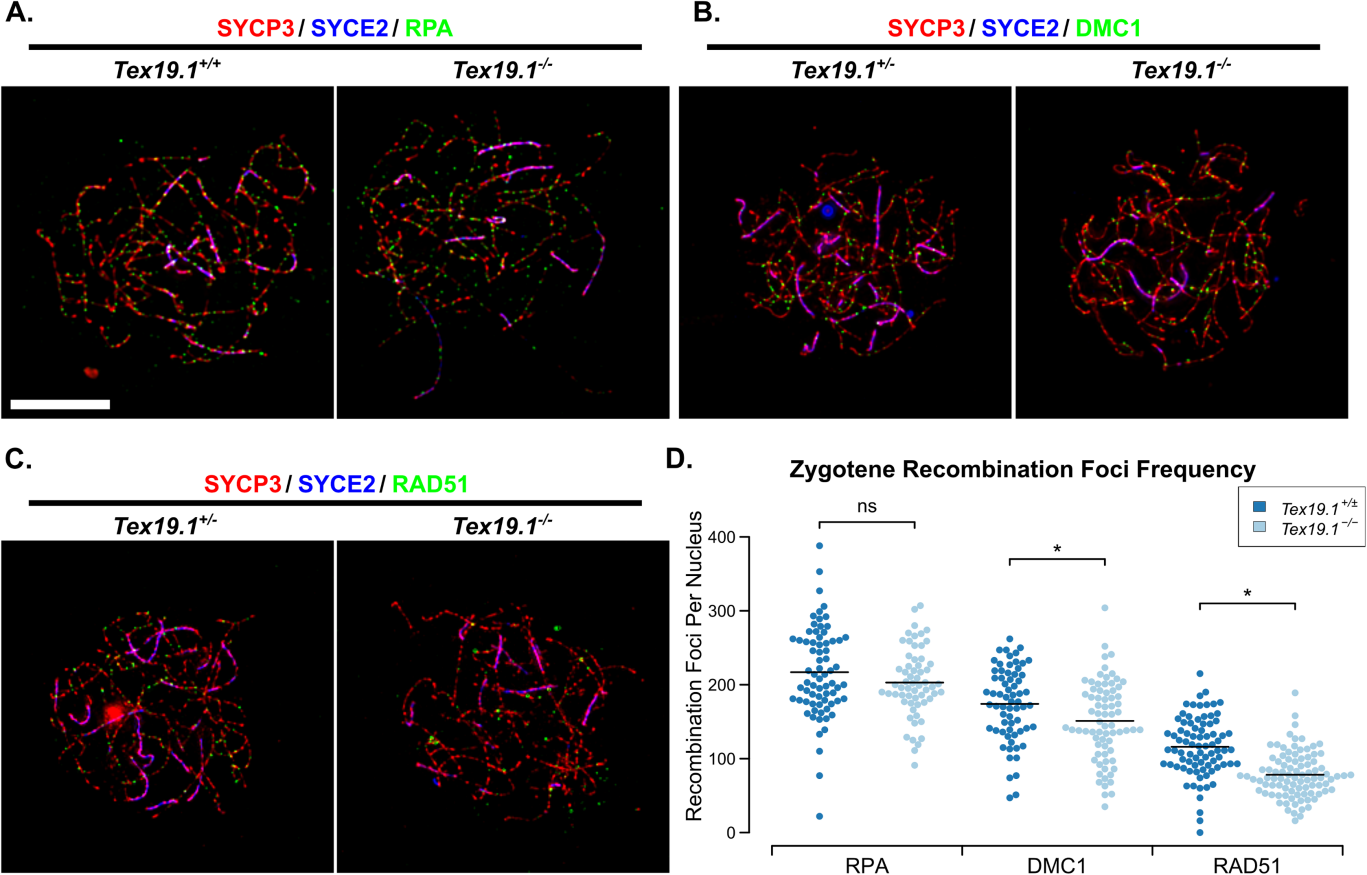
***Tex19*.*1*^*-/-*^ Spermatocytes Have Reduced Numbers of Recombination Foci During Zygotene.** (A-C) Immunostaining of chromosome spreads from *Tex19*.*1*^*+/±*^ and *Tex19*.*1*^*-/-*^ spermatocytes for the SC components SYCP3 (red) and SYCE2 (blue) to identify zygotene nuclei and chromosome axes, and RPA (A), DMC1 (B) and RAD51 (C) to mark recombination foci (green). Scale bar 10 μm. (D) Quantification of the number of RPA, DMC1 and RAD51 recombination foci in zygotene *Tex19*.*1*^*+/±*^ and *Tex19*.*1*^*-/-*^ spermatocytes. n = 71, 61, 66, 73, 81, 93 from three mice per genotype. Means are indicated with horizontal bars, * indicates p<0.01, and ns indicates no significant difference (Mann-Whitney U test). Control *Tex19*.*1*^*+/±*^ zygotene nuclei have 217±7 RPA, 174±6 DMC1, and 116±4 RAD51 foci; *Tex19*.*1*^*-/-*^ zygotene nuclei have 202±6 RPA, 151±6 DMC1, and 78±3 RAD51 foci.

We next investigated whether loss of *Tex19*.*1* impaired the abundance of recombination intermediates required for homologous chromosome pairing and synapsis. Chromosome spreads were immunostained for SYCP3, a component of the axial and lateral elements of the SC [31], and SYCE2, a component of the SC central element [32], to identify zygotene nuclei. Recombination foci associated with the chromosome axes were visualised by immunostaining for the single-stranded DNA binding proteins RPA, DMC1 and RAD51 [33]. The number of RPA, DMC1 and RAD51 foci in control *Tex19*.*1*^*+/±*^ zygotene nuclei (Fig 2) are all within the ranges previously reported for wild-type zygotene spermatocytes (150–250 for RPA foci, 100–250 for DMC1 and RAD51 foci) [33]. However, zygotene *Tex19*.*1*^*-/-*^ spermatocytes have fewer DMC1 and RAD51 foci than their littermate controls, with DMC1 and RAD51 foci frequency reduced to 87% and 67% of control levels respectively (Fig 2). Interestingly, the number of RPA foci is not statistically different from zygotene control nuclei (Fig 2), which could potentially reflect RPA foci being a later marker of recombination than RAD51 and DMC1 [34]. The differential behaviour of RAD51 and DMC1 foci in *Tex19*.*1*^*-/-*^ spermatocytes suggests that the generation, repair, or maturation kinetics of recombination foci is perturbed in the absence of *Tex19*.*1*.

**Fig 3:**
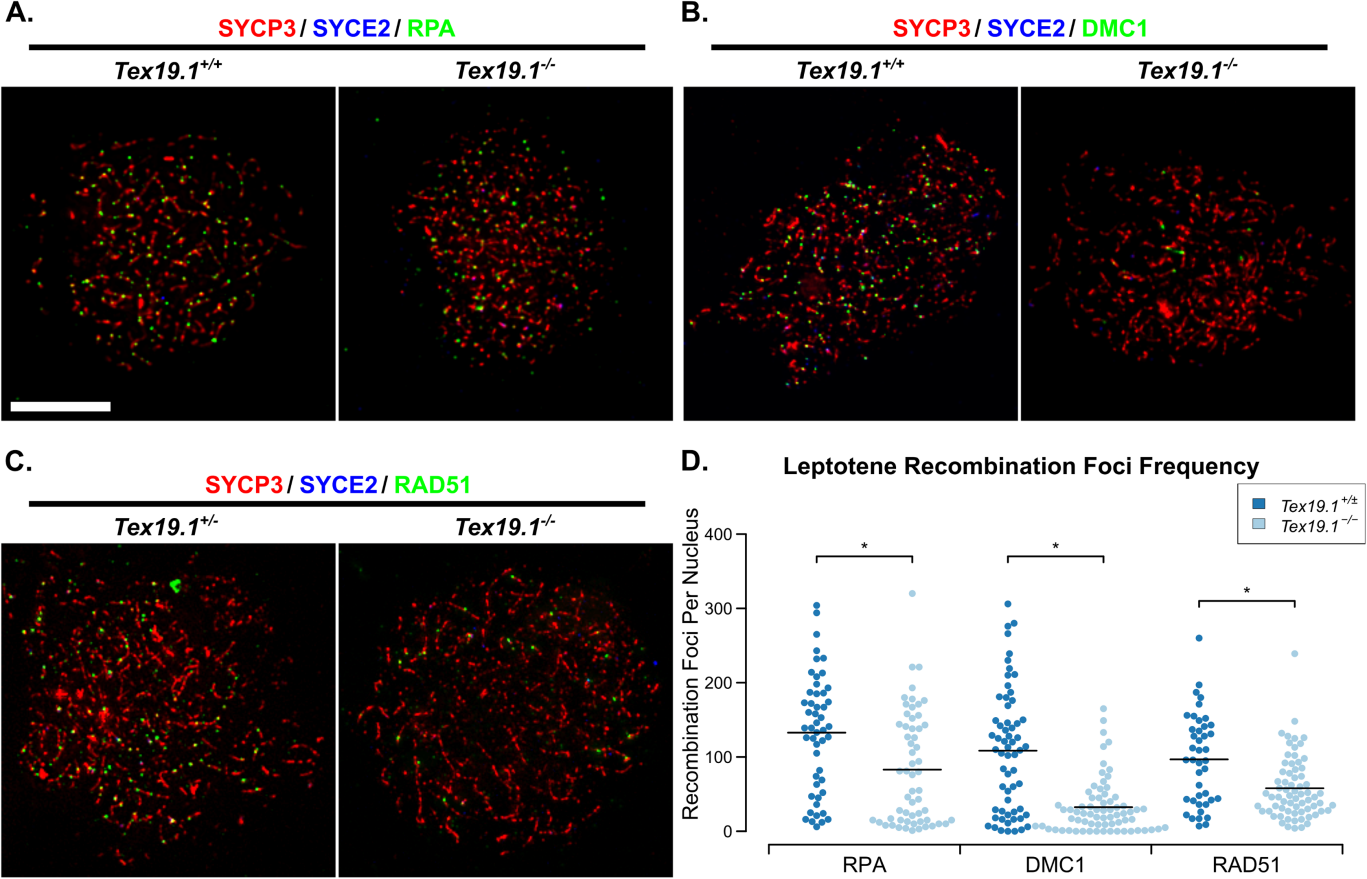
***Tex19*.*1*^*-/-*^ Spermatocytes Have Reduced Numbers of Recombination Foci During Leptotene.** (A-C) Immunostaining of chromosome spreads from *Tex19*.*1*^*+/±*^ and *Tex19*.*1*^*-/-*^ spermatocytes for the SC components SYCP3 (red) and SYCE2 (blue) to identify leptotene nuclei and fragments of chromosome axes, and RPA (A), DMC1 (B), and RAD51 (C) to mark recombination foci (green). Scale bar 10 μm. (D) Quantification of the number of RPA, DMC1 and RAD51-positive recombination foci in leptotene *Tex19*.*1*^*+/±*^ and *Tex19*.*1*^*-/-*^ spermatocytes. n = 50, 58, 61, 71, 42, 69 from three mice per genotype. Means are indicated with horizontal bars, and * indicates p<0.01 (Mann-Whitney U test). Control *Tex19*.*1*^*+/±*^ leptotene nuclei have 133±11 RPA, 108±11 DMC1, and 97±9 RAD51 foci; *Tex19*.*1*^*-/-*^ leptotene nuclei have 83±10 RPA, 32±4 DMC1, and 58±5 RAD51 foci.

Meiotic recombination is initiated during leptotene [9], therefore we next investigated whether loss of *Tex19*.*1* might perturb recombination foci frequency at this earlier stage of meiotic prophase. Counts of RPA, DMC1 and RAD51 foci in leptotene nuclei revealed a severe reduction in the frequency of each of these in the absence of *Tex19*.*1* (Fig 3). The numbers of RPA foci, DMC1 foci and RAD51 foci in leptotene *Tex19*.*1*^*-/-*^ spermatocytes were reduced to 63%, 30%, and 60% of those present in control spermatocytes (Fig 3). Thus, loss of *Tex19*.*1* results in reduced numbers of meiotic recombination foci in leptotene spermatocytes, a defect that precedes the chromosome asynapsis at pachytene.

The reduced numbers of recombination foci in *Tex19*.*1*^*-/-*^ spermatocytes could potentially decrease the efficiency of the DSB-dependent homology search and contribute to chromosome asynapsis in this mutant. Analysis of *Spo11* hypomorphs suggests that reduced numbers of meiotic DSBs impairs the initiation of synapsis and manifests as reduced numbers of SC fragments during late leptotene/early zygotene stages [14]. We therefore analysed the extent of synapsis in zygotene *Tex19*.*1*^*-/-*^ nuclei to assess whether the initiation of synapsis might similarly be impaired in these mutants. Chromosome spreads were immunostained with axial and central element SC markers and the percentage synapsis assessed in each zygotene nucleus (S1 Fig). In the absence of *Tex19*.*1*, most zygotene nuclei contained very low amounts of synapsis (<10%), whereas the majority of control zygotene nuclei contained intermediate levels of synapsis (10–70%, S1 Fig). The extent of synapsis in *Tex19*.*1*^*-/-*^ nuclei is more consistent with these mutants exhibiting a widespread block or delay in the initiation of synapsis throughout the nucleus, rather than defects in synapsis of specific chromosomes or progression of synapsis along the chromosome axes once it has initiated. Thus, as described for *Spo11* hypomorphs [14], the reduced numbers of recombination foci in *Tex19*.*1*^*-/-*^ sperrmatocytes during leptotene could potentially cause defects in homologous chromosome synapsis during zygotene resulting in asynapsis persisting in pachytene.

### *Tex19*.*1*^*-/-*^ spermatocytes have reduced amounts of *Spo11*-dependent recombination

**Fig 4:**
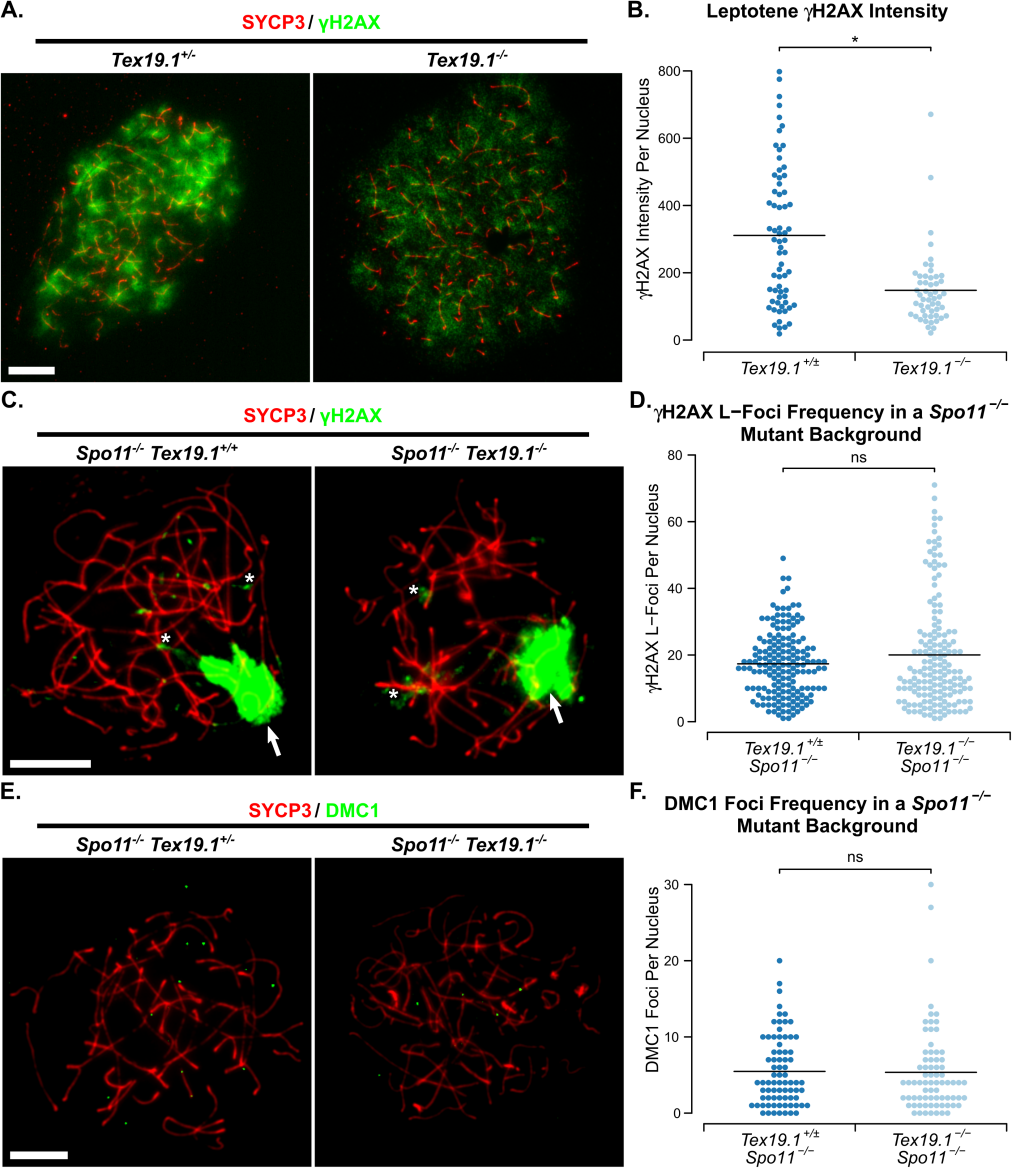
**Loss of *Tex19*.*1* Impairs the SPO11-Dependent Recombination in Spermatocytes.** (A) Immunostaining of chromosome spreads from *Tex19*.*1*^*+/±*^ and *Tex19*.*1*^*-/-*^ spermatocytes for the SC component SYCP3 (red) to identify leptotene nuclei and fragments of chromosome axes, and γH2AX (green) as a marker for DSBs. Scale bar 10 μm. (B) Quantification of γH2AX immunostaining intensity (arbitrary units) in leptotene *Tex19*.*1*^*+/±*^ and *Tex19*.*1*^*-/-*^ spermatocytes (311±15 and 148±26 units respectively). n = 66, 52 from three mice per genotype. Means are indicated with horizontal bars, and * indicates p<0.01 (Mann-Whitney U test). (C, E). Immunostaining of chromosome spreads from zygotene-like *Spo11*^*-/-*^ *Tex19*.*1*^*+/±*^ and *Spo11*^*-/-*^ *Tex19*.*1*^*-/-*^ spermatocytes for the SC component SYCP3 (red) to identify chromosome axes, and γH2AX (C, green), or DMC1 (E, green) as markers for DNA damage and recombination foci respectively. Arrows in C label the pseudo sex body, asterisks label example axis-associated L-foci. Scale bars 10 μm. D, F. Quantification of γH2AX L-foci (D) and DMC1-positive recombination foci (F) in zygotene-like *Spo11*^*-/-*^ *Tex19*.*1*^*+/±*^ and *Spo11*^*-/-*^ *Tex19*.*1*^*-/-*^ spermatocytes. n = 169, 162 for D; 76, 74 for F. Both analyses were performed on spreads from three mice per genotype. Means are indicated with horizontal bars, and ns indicates no significant difference (Mann-Whitney U test). *Spo11*^*-/-*^ *Tex19*.*1*^*+/±*^ and *Spo11*^*-/-*^ *Tex19*.*1*^*-/-*^ spermatocytes have 17.3±0.8 and 20.0±1.3 γH2AX L-foci; and 5.5±0.5 and 5.4±0.7 DMC1 foci respectively.

The reduced number of RPA, DMC1 and RAD51 foci in leptotene *Tex19*.*1*^*-/-*^ spermatocytes might reflect fewer *Spo11*-dependent DSBs in these cells, or defects in the processing and resection of those DSBs to form the single-stranded DNA ends that recruit RPA, DMC1 and RAD51, or accelerated repair of SPO11-induced DNA damage. Phosphorylation of the histone variant H2AX to generate γH2AX occurs in response to *Spo11*-dependent DSB formation [9], and is not impaired in spermatocytes proposed to be defective in subsequent processing of those DSBs [35]. We therefore tested whether loss of *Tex19*.*1* affects γH2AX abundance in leptotene spermatocytes. In both control and *Tex19*.*1*^*-/-*^ leptotene nuclei, γH2AX is present as a diffuse cloud of staining over regions of the nucleus (Fig 4A). Interestingly, quantification of the γH2AX signal showed that the amount of γH2AX in leptotene *Tex19*.*1*^*-/-*^ nuclei was around half that in *Tex19*.*1*^*+/±*^ controls (Fig 4B). Taken together, the reduced numbers of recombination foci and the reduced intensity of γH2AX immunostaining in *Tex19*.*1*^*-/-*^ spermatocytes suggests that loss of *Tex19*.*1* likely causes defects in early stages of *Spo11*-dependent recombination, or accelerated repair of SPO11-induced DNA damage.

The bulk of the γH2AX generated in spermatocytes reflects the generation of *Spo11*-dependent meiotic DSBs, however small amounts of γH2AX are generated independently of *Spo11* in these cells [9,36–39]. The extent of the decrease in γH2AX abundance in *Tex19*.*1*^*-/-*^ spermatocytes is arguably more consistent with reduced abundance of *Spo11*-dependent DSBs, but it is possible that loss of *Tex19*.*1* also affects *Spo11*-independent γH2AX generated during leptotene. To test directly whether loss of *Tex19*.*1* affects *Spo11*-independent γH2AX we quantified γH2AX abundance as well as DMC1 foci in *Spo11*^*-/-*^ *Tex19*.*1*^*-/-*^ double mutant spermatocytes. The relatively low levels of γH2AX present in *Spo11*^*-/-*^ spermatocytes typically manifests as a pseudo sex body, a cloud of γH2AX associated with a subset of asynapsed axes undergoing meiotic silencing of unsynapsed chromatin [38,39]. In addition to the pseudo sex body, smaller additional flares of chromosome axis-associated γH2AX staining termed L-foci are also present [36,37]. *Spo11*^*-/-*^ *Tex19*.*1*^*-/-*^ spermatocytes displayed similar γH2AX staining patterns and similar numbers of γH2AX L-foci as *Spo11*^*-/-*^ *Tex19*.*1*^*+/±*^ controls (Fig 4C, Fig 4D). Thus, pseudo sex body formation and *Spo11*-independent γH2AX L-foci frequency are independent of *Tex19*.*1*. In addition, although loss of *Tex19*.*1* impairs DMC1 foci frequency in a wild-type *Spo11* background (Fig 2, Fig 3), loss of *Tex19*.*1* has no detectable effect on DMC1 foci frequency in a *Spo11*^*-/-*^ mutant background (Fig 4E, Fig 4F). Thus, loss of *Tex19*.*1* appears to reduce the amount of *Spo11*-dependent recombination present in spermatocytes. In this respect the *Tex19*.*1*^*-/-*^ phenotype bears some resemblance to hypomorphic *Spo11* mutants [14,40]

Mutations in the genome-defence gene *Mael* have been reported to result in the accumulation of large amounts of *Spo11*-independent DNA damage as assessed by γH2AX staining and the presence of axis-associated RAD51 foci in late zygotene *Mael*^*-/-*^ *Spo11*^*-/-*^ double mutant spermatocytes [16]. However, in contrast to *Mael*^*-/-*^ *Spo11*^*-/-*^ double mutants [16], zygotene-like *Tex19*.*1*^*-/-*^ *Spo11*^*-/-*^ double mutant spermatocytes do not accumulate γH2AX (Fig 4C, Fig 4D) or axis-associated RAD51 foci (S2 Fig). Therefore, *Tex19*.*1* and the piRNA pathway component *Mael* appear to have different effects on *Spo11*-independent DNA damage in meiotic spermatocytes.

SPO11 is locally regulated in the nucleus, and feedback controls are thought to allow SPO11 to continue to generate DSBs on asynapsed regions of the chromosomes in late zygotene [14]. *Spo11* hypomorphs are still able to generate DSBs on asynapsed chromatin [14]. To assess whether asynapsed chromatin is similarly able to accumulate high levels of DSBs in *Tex19*.*1*^*-/-*^ mutants, we counted the number of RPA foci associated with the sex chromosomes, which remain largely asynapsed during pachytene. In the population of pachytene *Tex19*.*1* spermatocytes that successfully synapse all their autosomes, sex chomosomes were still able to accumulate similar numbers of RPA foci as control pachytene nuclei (S1 Fig). Thus, loss of *Tex19*.*1* does not prevent the accumulation of RPA foci on asynapsed chromatin.

Loss of *Tex19*.*1* results in female subfertility as well as male infertility [24]. However, loss of *Tex19*.*1* has sexually dimorphic effects on progression through meiotic prophase, and in contrast to its effects on spermatocytes, loss of *Tex19*.*1* does not cause defects in chromosome synapsis in female meiosis [26]. Nevertheless, it is possible that loss of *Tex19*.*1* could still cause a reduction in the number of early recombination foci in female meiosis that might not be sufficient to result in chromosome asynapsis. We therefore analysed RAD51 foci in E14.5 *Tex19*.*1*^*-/-*^ foetal oocytes to test whether loss of *Tex19*.*1* affects recombination in female meiosis. However, the number of RAD51 foci in late leptotene *Tex19*.*1*^*-/-*^ oocytes is not significantly different from late leptotene *Tex19*.*1*^*+/±*^ littermate controls (S3 Fig). Therefore *Tex19*.*1* is not required for accumulation of RAD51 foci in female meiosis and has a sexually dimorphic role in early meiotic recombination.

### Loss of *Tex19*.*1* does not impair MEI4 localisation or H3K4me3 deposition at recombination hotspots

**Fig 5:**
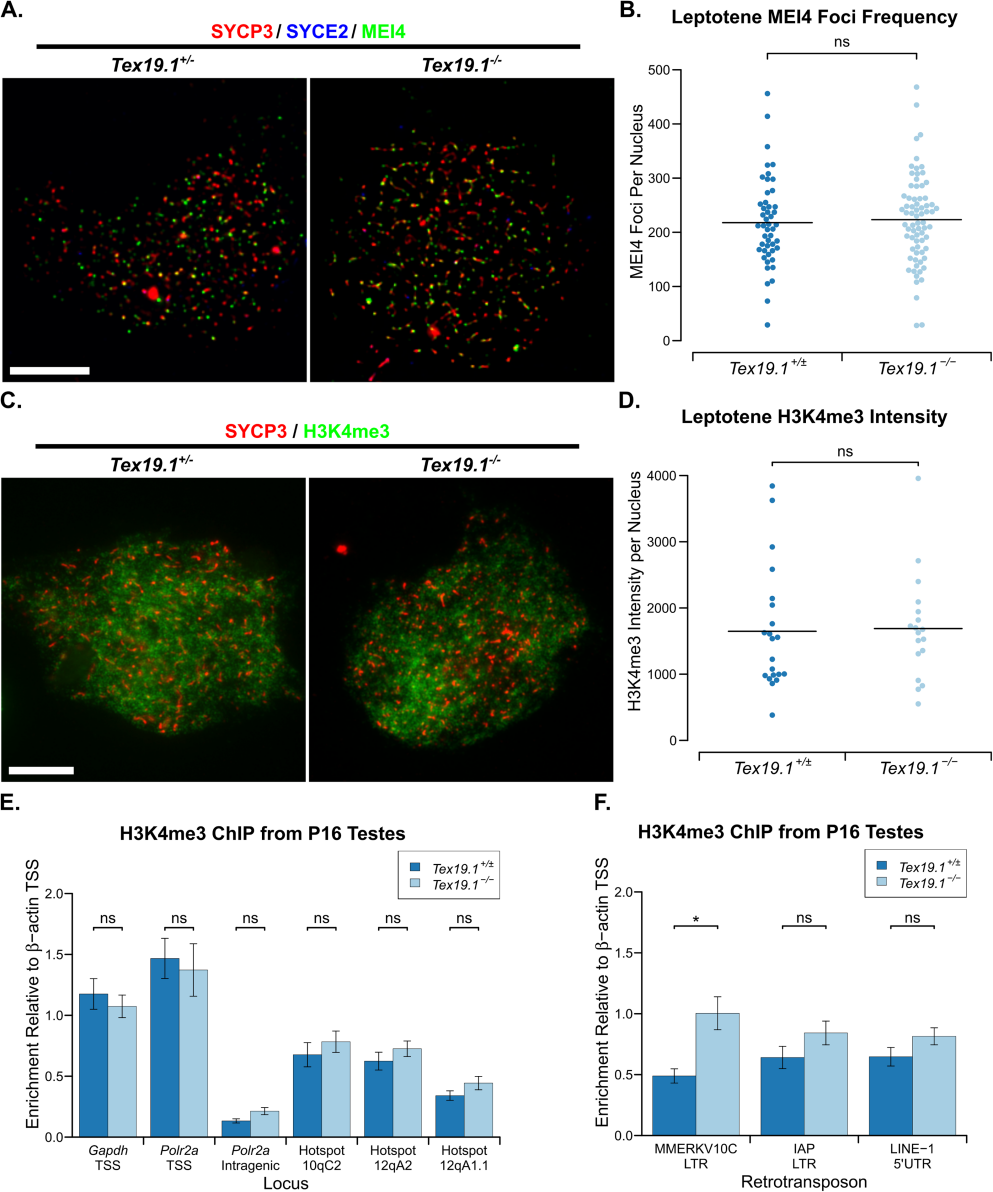
***Tex19*.*1*^*-/-*^ Spermatocytes Have No Overt Defects in MEI4 Localisation or in H3K4me3 Accumulation at Recombination Hotspots.** (A) Immunostaining of spermatocyte chromosome spreads for the SC components SYCP3 (red) to identify leptotene nuclei and fragments of chromosome axes, and MEI4 (green). Scale bar 10 μm. (B) Quantification of MEI4 foci in leptotene spermatocytes (218±12 for *Tex19*.*1*^*+/±*^, 223±10 for *Tex19*.*1*^*-/-*^, n = 48, 72 from three mice per genotype). Means are indicated with horizontal bars, ns indicates no significant difference (Mann-Whitney U test). (C) Immunostaining of spermatocyte chromosome spreads for the SC components SYCP3 (red) and SYCE2 (blue) to identify leptotene nuclei, and H3K4me3 (green). Scale bar 10 μm. (D) Quantification of anti-H3K4me3 staining intensity (1648±202 and 1689±187 arbitrary units respectively, n = 21, 18 from three mice per genotype). Means are indicated with horizontal bars, ns indicates no significant difference (Mann-Whitney U test). (E, F) H3K4me3 chromatin immunoprecipitation (ChIP) from P16 *Tex19*.*1*^*+/±*^ and *Tex19*.*1*^*-/-*^ testes. qPCR for recombination hotspots (E) and retrotransposon sequences (F) was performed on H3K4me3 ChIP and abundance measured relative to input chromatin, then normalised to enrichment for the β-actin (*Actb*) transcriptional start site (TSS). Mean normalised enrichment ± standard error from three animals of each genotype is shown. *Polr2a* and *Gapdh* TSSs were used as positive controls, and an intragenic region of *Polr2a* as a negative control. ns indicates no significant difference, * indicates p<0.05 (Student's t-test).

We next investigated whether the reduced frequency of *Spo11*-dependent recombination foci in leptotene *Tex19*.*1*^*-/-*^ spermatocytes might reflect defects upstream of *Spo11* in meiotic recombination. The requirements upstream of *Spo11* for meiotic DSB formation are relatively poorly understood in mammals, however SPO11 activity likely depends on the recruitment of the conserved axis-associated protein MEI4 to the chromosomal axes in leptotene [41]. We therefore quantified MEI4 foci in leptotene *Tex19*.*1*^*-/-*^ nuclei to test whether this event is perturbed by loss of *Tex19*.*1*. Control leptotene *Tex19*.*1*^*+/±*^ spermatocytes possess an average of 218 axis-associated MEI4 foci (Fig 5A, Fig 5B), similar but slightly lower than the average 309 foci per leptotene nucleus reported previously [41]. Leptotene *Tex19*.*1*^*-/-*^ nuclei possess similar numbers of MEI4 foci to leptotene *Tex19*.*1*^*+/±*^ controls (Fig 5A, Fig 5B). Thus, the reduced frequency of recombination foci seen in *Tex19*.*1*^*-/-*^ leptotene spermatocytes appears to be a consequence of defects acting downstream or independently of MEI4 localisation to chromosome axes.

*Spo11* function is also influenced by the activity of the histone methyltransferase PRDM9, which targets SPO11 to recombination hotspots [2,4,5]. Mutations in *Prdm9* result in reduced anti-H3K4me3 immunostaining in P14 spermatocytes, a failure to enrich H3K4me3 at *Prdm9*-dependent recombination hotspots, a reduction in recombination foci during early prophase, and meiotic chromosome asynapsis [5,42,43]. We therefore tested whether loss of *Tex19*.*1* might impair *Prdm9* function by assessing anti-H3K4me3 immunostaining intensity in leptotene nuclei. However, we could not detect a difference in the amount of anti-H3K4me3 immunostaining between *Tex19*.*1*^*+/±*^ and *Tex19*.*1*^*-/-*^ leptotene nuclei (Fig 5C, Fig 5D). To test whether the distribution of H3K4me3 rather than its total abundance might be altered in the absence of *Tex19*.*1* we performed H3K4me3 chromatin immunoprecipitation (ChIP)-qPCR on P16 testes. H3K4me3 is enriched at transcriptional start sites (TSSs) of active genes in addition to meiotic recombination hotspots [44], and as expected both *Tex19*.*1*^*+/±*^ and *Tex19*.*1*^*-/-*^ testes show enrichment of H3K4me3 at *Gapdh* and *Polr2a* active TSSs, but not at a *Polr2a* intragenic region (Fig 5E). However, loss of *Tex19*.*1* does not perturb the accumulation of H3K4me3 at *Prdm9*-dependent recombination hotspots (Fig 5E). Thus, the defects in *Spo11*-dependent recombination seen in *Tex19*.*1*^*-/-*^ spermatocytes does not appear to be a downstream consequence of impaired *Prdm9* activity.

*Tex19*.*1* plays a role in repressing retrotransposons in testes and placenta [21,24,45], and *Tex19*.*1*^*-/-*^ testes have increased abundance of *MMERVK10C* retrotransposon RNA, but not RNAs encoding *IAP* or *LINE-1* retrotransposons [24,45]. To test if the increase in *MMERVK10C* RNA is a consequence of transcriptional de-repression we also analysed retrotransposon sequences in the P16 testis H3K4me3 ChIP. Interestingly, the LTR driving *MMERVK10C* expression, but not *IAP* LTRs or *LINE-1* 5' UTR sequences are enriched in anti-H3K4me3 ChIP from *Tex19*.*1*^*-/-*^ testes relative to *Tex19*.*1*^*+/±*^ controls (Fig 5F). Thus the increase in *MMERVK10C* retrotransposon RNA abundance previously reported in *Tex19*.*1*^*-/-*^ testes [24,45] reflects, at least in part, transcriptional de-repression of this element. However, the 2-fold increase in H3K4me3 abundance at *MMERVK10C* LTR sequences does not detectably interfere or compete with enrichment of H3K4me3 at *Prdm9*-dependent recombination hotspots.

### The *Tex19*.*1*^*-/-*^ meiotic recombination defect is phenocopied by mutations in *Ubr2*

**Fig 6:**
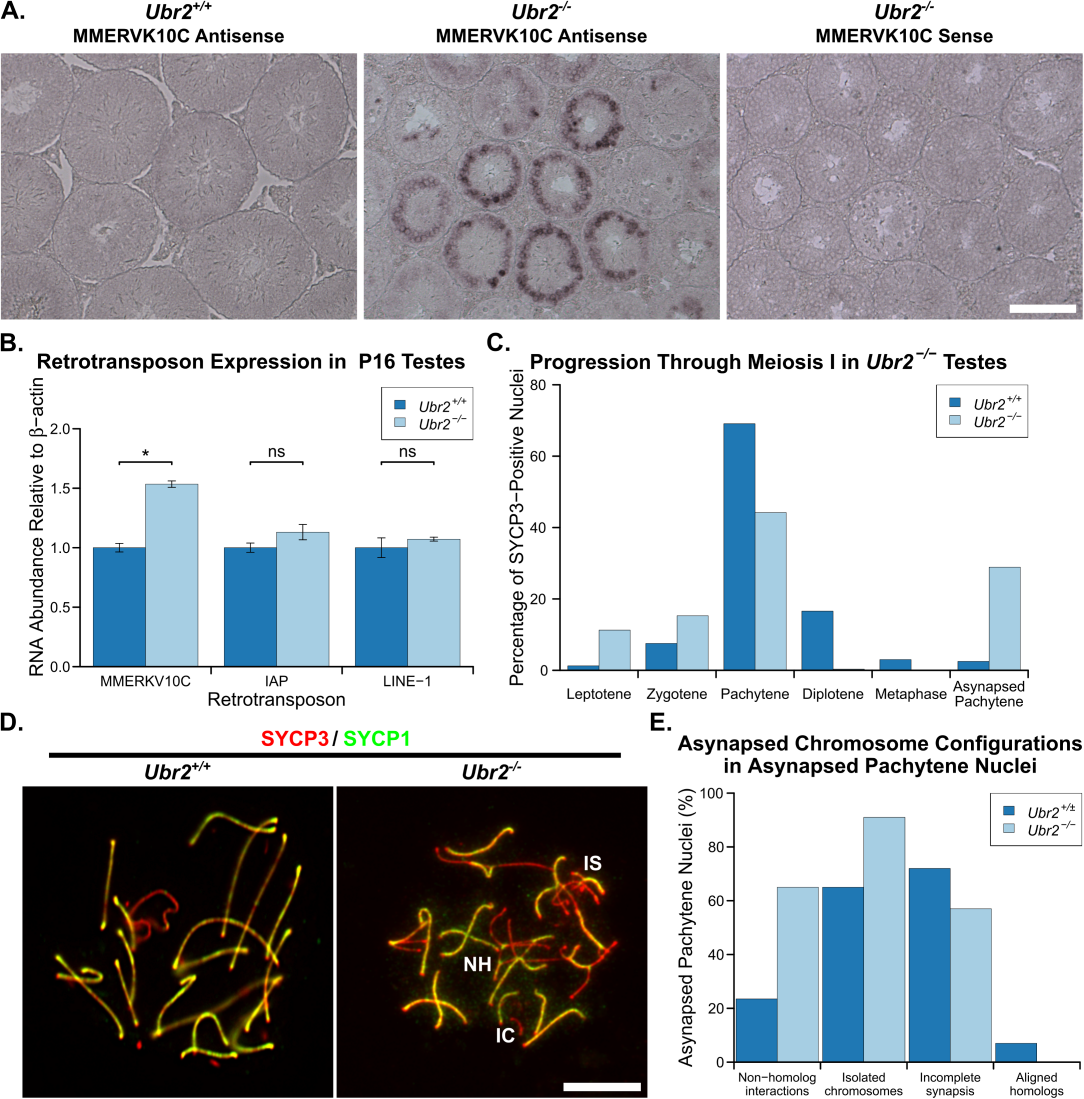
***Ubr2*^*-/-*^ Spermatocytes Phenocopy the Retrotransposon Derepression and Asynapsis Phenotypes Present in *Tex19*.*1*^*-/-*^ Mutants.** (A) In situ hybridisation for *MMERVK10C* retrotransposon RNA in *Ubr2*^*-/-*^ testes. Specific signal (dark purple precipitate) is present in *Ubr2*^*-/-*^ spermatocytes hybridised with an antisense *MMERVK10C* probe. Scale bar 100 μm. (B) qRT-PCR for *MMERVK10C*, *IAP* and *LINE-1* retrotransposons in P16 *Ubr2*^*-/-*^ testes. Mean abundance of retrotransposon RNAs relative to β-actin is shown for two *Ubr2*^*+/+*^ and three *Ubr2*^*-/-*^ animals. ns indicates no significant difference, * indicates p<0.05 (Student's t-test). (C) Chromosome spreads from testes from three *Ubr2*^*+/+*^ and three *Ubr2*^*-/-*^ animals were immunostained with antibodies to SYCP3 and SYCP1, and SYCP3-positive nuclei scored for meiotic substage (n = 398, 595). *Ubr2*^*-/-*^ spreads contained a significant proportion of aberrant pachyene nuclei containing asynapsed chromosomes, but few diplotene or metaphase I nuclei compared to *Ubr2*^*+/+*^ controls. (D) Immunostaining of chromosome spreads from *Ubr2*^*+/+*^ and *Ubr2*^*-/-*^ spermatocytes for SYCP3 (red) and SYCP1 (green). Asynapsed chromosomes assemble SYCP3 but not SYCP1, and examples involving non-homologous interactions (NH), incomplete synapsis between homologs (IS), and isolated chromosomes (IC) are labelled. Scale bar 10 μm. (E) Percentage of asynapsed pachytene *Ubr2*^*+/+*^ and *Ubr2*^*-/-*^ spermatocytes exhibiting the indicated categories of asynapsed chromosomes (n = 17, 99 respectively from a total of three *Ubr2*^*+/+*^ and three *Ubr2*^*-/-*^ mice). Each nucleus is typically represented in more than one category. Fully autosomally synapsed pachytene nuclei are not included in this analysis, and only asynapsed pachytene nuclei containing clearly distinguishable asynapsed chromosome configurations were scored.

TEX19.1 physically interacts with the E3 ubiquitin ligase UBR2 [25] and regulates its activity [26,27]. TEX19.1 protein is undetectable in *Ubr2*^*-/-*^ testes, suggesting that much of the TEX19.1 protein in the testis requires UBR2 for its stability [25]. *Ubr2* is implicated in the ubiquitylation and degradation of N-end rule substrates and previous reports suggest that loss of *Ubr2* causes variable defects in spermatogenesis possibly depending on the strain background [46]. Some *Ubr2*^*-/-*^ spermatocytes are reported to progress into pachytene and arrest due to defects in the accumulation of ubiquitylated histone H2A at the sex body and meiotic sex chromatin inactivation during pachytene [47,48]. *Ubr2*^*-/-*^ spermatocytes are also reported to arrest and apoptose in prophase I due to defects in the repair of DSBs, homologous chromosome pairing, and SC formation [46,48]. Given the lack of detectable TEX19.1 protein in *Ubr2*^*-/-*^ testes, we tested whether the reported defects in homologous chromosome pairing and SC formation in *Ubr2*^*-/-*^ spermatocytes [46] might reflect earlier defects in the initiation of meiotic recombination similar to *Tex19*.*1*^*-/-*^ spermatocytes. We generated *Ubr2*^*-/-*^ mice carrying a premature stop codon in the N-terminal region of UBR2 within the UBR domain that binds N-end rule substrates. The *Ubr2*^*-/-*^ mice analysed here have no detectable UBR2 protein in their testes (S4 Fig), a 68% reduction in testis weight (S4 Fig), and no detectable sperm in their epididymis (S4 Fig), consistent with *Ubr2*^*-/-*^ spermatogenesis defects reported previously [46]. The seminiferous tubules in *Ubr2*^*-/-*^ mice contain reduced numbers of post-meiotic round and elongated spermatids, and accumulations of pyknotic and zygotene-like nuclei consistent with meiotic defects (S4 Fig) as reported previously [46,48]. Similar to *Tex19*.*1*^*-/-*^ testes [24], some round and elongated post-meiotic spermatids are detectable in *Ubr2*^*-/-*^ testes suggesting that any meiotic defects present do not completely block spermatogenesis. Furthermore, loss of *Ubr2* phenocopies the specific retrotransposon derepression seen in *Tex19*.*1*^*-/-*^ testes [24]: *MMERVK10C*, but not *LINE-1* or *IAP*, retrotransposon RNAs are derepressed in *Ubr2*^*-/-*^ spermatocytes (Fig 6A, Fig 6B).

**Fig 7:**
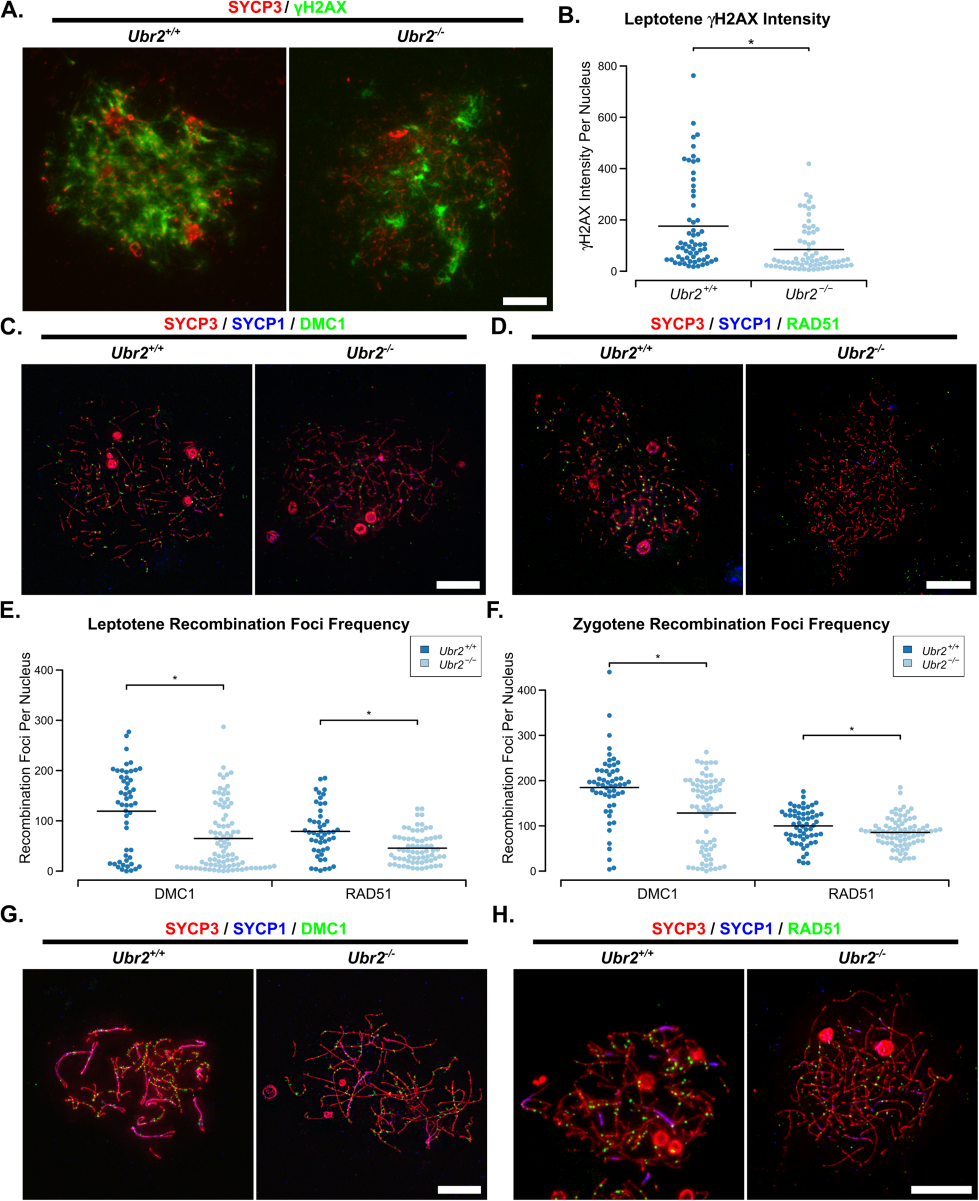
***Ubr2*^*-/-*^ Spermatocytes Have Defects in Early Meiotic Recombination.** (A) Immunostaining of chromosome spreads from *Ubr2*^*+/+*^ and *Ubr2*^*-/-*^ spermatocytes for the SC component SYCP3 (red) to identify leptotene nuclei and fragments of chromosome axes, and γH2AX (green) as a marker for DSBs. Scale bar 10 μm. (B) Quantification of γH2AX immunostaining intensity (arbitrary units) in leptotene *Ubr2*^*+/+*^ and *Ubr2*^*-/-*^ spermatocytes (176±23 and 85±11 units respectively). n = 60, 68 from three mice per genotype. Means are indicated with horizontal bars, and * indicates p<0.01 (Mann-Whitney U test). (C, D, G, H) Immunostaining of chromosome spreads from *Ubr2*^*+/+*^ and *Ubr2*^*-/-*^ spermatocytes for the SC components SYCP3 (red) and SYCP1 (blue) to identify leptotene nuclei (C, D), zygotene nuclei (G, H), and fragments of chromosome axes; and either DMC1 (C, G, green) or RAD51 (D, H, green) to mark recombination foci. Scale bar 10 μm. (E, F) Quantification of the number of DMC1-positive and RAD51-positive recombination foci in spermatocytes from two *Ubr2*^*+/+*^ and three *Ubr2*^*-/-*^ spermatocytes during leptotene (E) and zygotene (F). Means are indicated with horizontal bars, and * indicates p<0.05 (Mann-Whitney U test). Control *Ubr2*^*+/+*^ nuclei have 124±13 DMC1 foci in leptotene (n = 40) and 194±9 in zygotene (n = 52); *Ubr2*^*-/-*^ nuclei have 65±7 DMC1 foci in leptotene (n = 89) and 128±9 in zygotene (n = 75). Control *Ubr2*^*+/+*^ nuclei have 79±7 RAD51 foci in leptotene (n = 48) and 100±5 in zygotene (n = 59); *Ubr2*^*-/-*^ nuclei have 46±4 RAD51 foci in leptotene (n = 70) and 85±4 in zygotene (n = 82).

We tested whether the meiotic defects in *Ubr2*^*-/-*^ spermatocytes might resemble the asynapsis seen in *Tex19*.*1*^*-/-*^ spermatocytes (Fig 1A and 1B). Chromosome spreads from *Ubr2*^*-/-*^ testes confirm that this *Ubr2* mutant allele causes defects in progression through meiotic prophase, and very few spermatocytes progress through pachytene into diplotene (Fig 6C). Furthermore, around 40% of pachytene *Ubr2*^*-/-*^ spermatocytes had at least one asynapsed autosome pair when staging SYCP3-positive nuclei for meiotic progression under low magnification (Fig 6C, Fig 6D). At higher magnification, 65.9% ± 2.5 *Ubr2*^*-/-*^ pachytene nuclei from three animals have some autosomal asynapsis, compared to 11.3% ± 2.5 pachytene nuclei from three *Ubr2*^*+/+*^ animals (p<0.001, Student’s t-test). Like in *Tex19*.*1*^*-/-*^ spermatocytes (Fig 1A, Fig 1B), these asynapsed chromosomes are present in multiple configurations consistent with defects in recombination or homolog pairing (Fig 6E). Similar to *Tex19*.*1*^*-/-*^ spermatocytes, the asynapsis in *Ubr2*^*-/-*^ spermatocytes is also associated with earlier defects in meiotic recombination. γH2AX abundance, DMC1 foci frequency and RAD51 foci frequency are reduced to around 50%, 52% and 58% respectively of those seen during leptotene in *Ubr2*^*-/-*^ mutants (Fig 7), which contrasts with a previous report that γH2AX staining, and RAD51 and RPA foci frequency are unaffected in leptotene *Ubr2*^*-/-*^ spermatocytes [48]. Consistent with the decrease in leptotene recombination foci frequency reported here, DMC1 and RAD51 foci frequency remain around 66% and 86% respectively of that seen in control spermatocytes during zygotene (Fig 7). As there appeared to be some qualitative similarity between the defects in recombination foci frequency in *Ubr2*^*-/-*^ and *Tex19*.*1*^*-/-*^ spermatocytes, we tested whether this meiotic recombination defect would be sufficient to delay or impair the initiation of chromosome synapsis in *Ubr2*^*-/-*^ spermatocytes. Measurement of the extent of chromosome synapsis in zygotene *Ubr2*^*-/-*^ spermatocytes suggests that, like in *Tex19*.*1*^*-/-*^ mutants (S1 Fig) and *Spo11* hypomorphs [14], synapsis is delayed in the absence of *Ubr2* (S4 Fig). These data suggest that the defect in progression to pachytene previously reported in *Ubr2*^*-/-*^ mutants [46] may reflect loss of TEX19.1 protein and earlier defects in the meiotic recombination in these mutants. Furthermore, these data show that *Ubr2* and *Tex19*.*1* are both required to allow sufficient early recombination foci to accumulate to drive robust homologous chromosome synapsis in mouse spermatocytes.

## Discussion

### Meiotic recombination and chromosome synapsis in meiosis

This study aimed to elucidate the mechanistic basis of the chromosome synapsis defect in male mice carrying mutations in the germline genome defence gene *Tex19*.*1* [24]. We have shown that the pachytene chromosome asynapsis in these mice, and in mice carrying mutations in the TEX19.1-interacting protein UBR2, is likely a downstream consequence of reduced meiotic recombination earlier in meiotic prophase. Wild-type mice generate around 10-fold more meiotic DSBs than there are chiasmata, and the large numbers of DSBs generated in leptotene and zygotene appear to be important to drive pairing and synapsis of homologous chromosomes [2,7,8]. Allelic series of *Spo11* activity suggest that reducing the number of meiotic DSBs to around 50% of normal levels is sufficient to cause chromosome asynapsis [14,40]. The reduction in early recombination foci seen in leptotene *Tex19*.*1*^*-/-*^ and leptotene *Ubr2*^*-/-*^ spermatocytes, is similar to this threshold and could be sufficient to account for the chromosome asynapsis seen in these mutants. Notably, *Tex19*.*1*^*-/-*^ spermatocytes and *Ubr2*^*-/-*^ spermatocytes do not exhibit a severe asynapsis phenotype: only a proportion of pachytene *Tex19*.*1*^*-/-*^ or *Ubr2*^*-/-*^ spermatocytes have asynapsed chromosomes, and there is some progression to post-meiotic spermatid stages in both these mutants. Thus the ~50% reduction in leptotene DSB frequency caused by loss of *Tex19*.*1* or *Ubr2* could be sufficient to cause the level of asynapsis present in these spermatocytes.

Interestingly, the frequency of recombination foci in zygotene *Tex19*.*1*^*-/-*^ spermatocytes is closer to wild-type levels than that seen during leptotene, suggesting additional recombination foci are accumulating during zygotene that allow the *Tex19*.*1*^*-/-*^ spermatocytes to catch up with wild-type cells. It is possible that DSB generation is delayed in *Tex19*.*1*^*-/-*^ spermatocytes, or that repair of DSBs is accelerated in leptotene but not zygotene *Tex19*.*1*^*-/-*^ spermatocytes, or that this compensation of the *Tex19*.*1* recombination deficiency during zygotene reflects control mechanisms that regulate DSB frequency in meiotic cells [14]. An overall delay in germ cell development is probably not causing a delay in meiotic recombination relative to axial element assembly as previous analysis of gene expression profiles in P16 *Tex19*.*1*^*-/-*^ testes does not exhibit enrichment of genes expressed in more immature germ cells such as spermatogonia or leptotene spermatocytes [24,45]. In hypomorphic *Spo11* mice, DSBs are generated on asynapsed regions of the chromosomes during zygotene, potentially stimulating homology search and synapsis in these regions [14]. However, although any additional early recombination foci that accumulate in zygotene in *Tex19*.*1*^*-/-*^ spermatocytes might be rescuing asynapsis to some degree, they are not sufficient to allow the majority of *Tex19*.*1*^*-/-*^ spermatocytes to complete synapsis.

### Meiotic defects in genome defence mutants

*Tex19*.*1* is one of a group of germline genome defence genes which cause retrotransposon de-repression and defects in meiotic chromosome synapsis [15]. Although a common mechanism could link de-repression of retrotransposons and chromosome asynapsis in these mutants, mutations in different germline genome defence genes seem to have distinct effects on DNA damage and recombination during meiosis. Mutations in *Mael* cause a striking increase in *Spo11*-independent DNA damage in meiotic spermatocytes which has been proposed to represent DSBs generated by retrotransposon-encoded endonucleases [16], but the absence of any detectable increase in *Spo11*-independent DNA damage in *Spo11*^*-/-*^ *Tex19*.*1*^*-/-*^ spermatocytes reported here contrasts markedly with the phenotype of *Spo11*^*-/-*^ *Mael*^*-/-*^ spermatocytes. Moreover, zygotene recombination foci frequency is reduced in *Tex19*.*1*^*-/-*^ spermatocytes, but are not perturbed by mutations in *Dnmt3l* [18,19]. Although *Tex19*.*1* has been proposed to be part of the piRNA pathway [49], the phenotypic differences between the meiotic defects in *Tex19*.*1* mutants and different germline genome defence mutants, indicate that distinct mechanisms may be causing asynapsis in each of these mutants.

The spectrum of retrotransposons de-repressed in *Tex19*.*1*^*-/-*^ spermatocytes differs from those de-repressed in *Mael*^*-/-*^ testes and *Dnmt3l*^*-/-*^ testes [16,17,24,45]. It is therefore possible that some of the differences between the meiotic phenotypes of these mutants reflects differences in the type of retrotransposon de-repressed or the mechanism of de-repression. Data from *Dnmt3l*^*-/-*^ mice suggests that transcriptional activation of *LINE-1* retrotransposons alters the distribution of meiotic recombination foci and induces recombination at *LINE-1* elements leading to interactions between non-homologous chromosomes [19]. It is not clear if transcriptional activation of *MMERVK10C* elements in *Tex19*.*1*^*-/-*^ spermatocytes causes a similar re-distribution of meiotic recombination foci. Neither is it clear if loss of *Tex19*.*1* perturbs recombination at all meiotic recombination hotspots equally. Thus, we cannot rule out the possibility that altered distribution of meiotic recombination is contributing to chromosome asynapsis in *Tex19*.*1*^*-/-*^ spermatocytes. However, recombination foci abundance in *Tex19*.*1*^*-/-*^ spermatocytes is reduced to a level similar to that seen in hypomorphic *Spo11* mutants, which also have defects in chromosome synapsis [14,40]. Thus, reduced meiotic recombination is likely the primary cause of chromosome asynapsis in *Tex19*.*1*^*-/-*^ spermatocytes.

### Roles for *Tex19*.*1* and *Ubr2* in meiotic recombination

The data presented here suggest that both *Tex19*.*1* and *Ubr2* are required for sufficient meiotic recombination to drive robust chromosome synapsis in spermatocytes. We have shown defects in the number of early recombination foci, and in the amount of γH2AX present during leptotene in both these mutants. Further experiments are required to delineate which stage in early recombination is disrupted in these mutants. It is possible that SPO11 activity and DSB formation itself is reduced or delayed. Alternatively early stages in processing SPO11-dependent DSBs or signalling the SPO11-induced DNA damage could be perturbed in these mutants. Or SPO11-dependent DSBs or recombination intermediates could be repaired more rapidly in the absence of *Tex19*.*1* or *Ubr2*. In contrast to males, *Tex19*.*1* was not required for synapsis in oocytes, or for the generation of normal numbers of RAD51 foci in female meiosis. This could reflect a difference in the genetic requirement for *Spo11*-dependent recombination between male and female meiosis, or alternatively could reflect some redundancy between *Tex19*.*1* and its paralog, *Tex19*.*2* at this stage of development [20,23].

UBR2 was previously suggested not to have a role in the initiation of meiotic recombination as it did not localise to recombination foci and was not required for normal recruitment of RAD51 or RPA to recombination foci during leptotene [48]. Immunocytologically-detectable enrichment at recombination foci is probably not a requirement for UBR2 to directly or indirectly influence the initiation of meiotic recombination. However, the effect of *Ubr2* on recombination foci and γH2AX during leptotene reported here does contradict the previous description of the *Ubr2*^*-/-*^ leptotene spermatocyte phenotype, although representative images and quantitative analysis of recombination foci in leptotene *Ubr2*^*-/-*^ spermatocytes were not shown in that study [48]. Differences between mouse strain background or *Ubr2* allele being studied may contribute to this, and the delay in synapsis initiation during zygotene (S4 Fig) could also complicate meiotic prophase substaging during analysis of *Ubr2*^*-/-*^ spermatocytes leading to differences between studies. However, reduced numbers of recombination foci in zygotene *Ubr2*^*-/-*^ spermatocytes have been reported previously [48] and are consistent with the data presented here. Our data indicate that the reduction in zygotene recombination foci in *Ubr2*^*-/-*^ spermatocytes is a consequence of earlier defects in the meiotic recombination during leptotene, and that reduced meiotic recombination is contributing to the *Ubr2*^*-/-*^ meiotic phenotype.

The phenotypic similarity between *Tex19*.*1*^*-/-*^ mutants and *Ubr2*^*-/-*^ mutants, in combination with the physical interaction between TEX19.1 and UBR2 proteins [25], and the requirement for *Ubr2* for TEX19.1 protein stability [25], suggests that TEX19.1 and UBR2 are functioning in the same pathway to promote meiotic recombination. However, the molecular mechanism underlying the genetic requirement for *Tex19*.*1* and *Ubr2* in meiotic recombination is not clear. It is possible that the defects in the initiation of meiotic recombination in *Ubr2*^*-/-*^ spermatocytes described here reflects the absence of TEX19.1 protein in these cells and uncharacterised downstream functions of TEX19.1 in promoting meiotic recombination. Alternatively, it is possible TEX19.1 is regulating the activity of UBR2 [26], a RING-domain E3 ubiquitin ligase, and that TEX19.1’s effects on meiotic recombination reflect a role for at least one of UBR2’s substrates in this process. Indeed, loss of *Tex19*.*1* might have effects on multiple UBR2 substrates and that could be responsible for the different aspects of the *Tex19*.*1*^*-/-*^ phenotype in different developmental stages and tissues. UBR2 has been shown to ubiquitylate histone H2A and histone H2B [47], and is implicated in degrading the C-terminal fragment of the REC8 cohesin subunit generated by separase-dependent cleavage [50]. Therefore it is possible that loss of *Tex19*.*1* or *Ubr2* affects meiotic chromosome organisation or the meiotic chromatin substrate on which the SPO11 endonuclease is acting. It is also possible that UBR2 ubiquitylates SPO11, or one of its regulators, and that loss of *Tex19*.*1* or *Ubr2* affects the amount of SPO11 activity rather than its chromatin substrate in leptotene spermatocytes. These possibilities are not mutually exclusive and further work is required to elucidate how the *Tex19*.*1*-*Ubr2* pathway influences meiotic recombination. However, the data presented here demonstrates that *Tex19*.*1* and *Ubr2* are genetically required to ensure that sufficient recombination is present in male meiosis.

## Materials and methods

### Ethics statement

Animal experiments were carried out under authority of UK Home Office Project Licence PPL 60/4424 after ethical approval by University of Edinburgh Animal Welfare and Ethical Review Body.

### Mice

*Tex19*.*1*^*-/-*^ animals backcrossed three times to a C57BL/6 genetic background were bred and genotyped as described [24]. *Spo11*^*+/-*^ heterozygous mice [7] on a C57BL/6 genetic background [18] were inter-crossed with *Tex19*.*1*^*+/-*^ mice. Animal experiments were carried out under UK Home Office Project Licence PPL 60/4424. Noon on the day that a plug was found was designated E0.5, day of birth was designated P1, and adult mice were typically analysed at between 6–14 weeks old. *Tex19*.*1*^*+/+*^ and *Tex19*.*1*^*+/-*^ animals have no difference in testis weights or sperm counts [24] and did not show any difference in leptotene recombination foci frequency (171 ± 7 and 185 ± 7 DMC1 foci; 217 ± 7 and 217 ± 8 RPA foci for wild-type and heterozygous mice respectively, no significant difference by Mann-Whitney U test, n = 40, 21, 10, 40 respectively). Therefore data from these control genotypes were pooled as *Tex19*.*1*^*+/±*^ to reduce animal use. Epididymal sperm counts were determined as described [24]. *Ubr2*^*-/-*^ mice were generated by CRISPR/Cas9 double nickase-mediated genome editing in zygotes [51]. Complementary oligonucleotides (S2 Table) targeting exon 3 of *Ubr2* were annealed and cloned into plasmid pX335 [52], amplified by PCR, then in vitro transcribed using a T7 Quick High Yield RNA Synthesis kit (NEB) to generate paired guide RNAs. RNA encoding the Cas9 nickase mutant (50 ng/µl, Tebu-Bio), paired guide RNAs targeting exon 3 of UBR2 (each at 25 ng/µl), and 150 ng/µl single-stranded DNA oligonucleotide repair template (S2 Table) were microinjected into the cytoplasm of C57BL/6 × CBA F2 zygotes. The repair template introduces an *Xba*I restriction site and mutates cysteine-121 within the UBR domain of *Ubr2* (Uniprot Q6WKZ8-1) to a premature stop codon. The zygotes were then cultured overnight in KSOM (Millipore) and transferred into the oviduct of pseudopregnant recipient females. Pups were genotyped and the mutant *Ubr2* allele back-crossed to C57BL/6.

### Immunostaining meiotic chromosome spreads

Chromosome spreads from testes were prepared as described by Peters et al. [53] for the *Spo11*^*-/-*^ and *Tex19*.*1*^*-/-*^ *Spo11*^*-/-*^ analyses, or by Costa et al. [32] for all other analyses. Chromosome spreads were prepared from foetal ovaries as described [54]. For immunostaining, slides were blocked and antibodies diluted in PBS containing 0.15% BSA, 0.1% Tween-20 and 5% goat serum as indicated in S1 Table. The anti-MEI4, anti-SYCE2, and anti-RPA primary antibodies used were as reported [41,54,55]. Alexa Fluor-conjugated secondary antibodies (Invitrogen) were used at a 1:500 dilution, and 2 ng/μl 4,6-diamidino-2-phenylidole (DAPI) was used to fluorescently stain DNA. Slides were mounted in 90% glycerol, 10% PBS, 0.1% p-phenylenediamine. Three or four channel images were captured with iVision or IPLab software (BioVision Technologies) using an Axioplan II fluorescence microscope (Carl Zeiss) equipped with motorised colour filters. Immunostaining was performed on spreads from at least three experimental and three control animals unless otherwise stated. Statistical analysis was performed in R [56], means are reported ± standard error, and n is reported as total number of spreads analysed in each experiment.

Nuclei were staged by immunostaining for the axial/lateral element marker SYCP3 [31]. Nuclei with short fragments of axial element but no synapsis were classified as leptotene, nuclei containing some regions of axial element undergoing synapsis along with some regions of axial element not undergoing synapsis were classified as zygotene, and those with complete autosomal synapsis as pachytene. Immunostaining for the central element component SYCE2 [32], or the transverse filament component SYCP1 [57], were included in some experiments to monitor synapsis. Asynapsed pachytene nuclei [24] were identified due to the presence of at least one completely synapsed pair of autosomes and at least one incompletely synapsed pair of autosomes exhibiting asynapsis along at least half its length. Nuclei from *Spo11*^*-/-*^ and *Spo11*^*-/-*^ *Tex19*.*1*^*-/-*^ mice that had complete axial element formation were classified as zygotene-like regardless of the extent of synapsis. For analysis of RAD51 foci in late leptotene oocytes in chromosome spreads from E14.5 foetal ovaries, oocytes with extensive linear SYCP3 staining, indicating axial element formation, and an absence of clear interactions between these axes, were judged to be in late leptotene.

Recombination foci in leptotene and zygotene nuclei were imaged by capturing z-stacks using a piezoelectrically-driven objective mount (Physik Instrumente) controlled with Volocity software (PerkinElmer). These images were deconvolved using Volocity, a 2D image generated in Fiji [58], and analysed in Adobe Photoshop CS6. DMC1, RAD51 and RPA foci were counted as recombination foci when they overlapped a chromosome axis. To measure leptotene γH2AX or H3K4me3 signal intensity, nuclear area was delimited using the DAPI signal, and signal intensity in that area quantified and corrected for background non-nuclear signal in 16 bit grayscale images using Fiji software. To assess the extent of synapsis in zygotene nuclei (S1 Fig, S4 Fig), the total length of completely assembled SC was estimated by SYCP1 or SYCE2 staining and expressed relative to the total length of SYCP3-containing axial/lateral element in that nucleus. For this and all immunocytological scoring, images were scored blind with respect to genotype by pooling control and knockout images, randomly assigning new filenames to each image, then decoding the filenames after scoring.

### Chromatin immunoprecipitation (ChIP)

Decapsulated P16 testes were macerated with razor blades in ice-cold PBS, tissue fragments were removed by allowing to settle, and testicular cells pelleted at 860*g* for 5 minutes at 4°C. The cells were resuspended in PBS and cross-linking ChIP performed essentially as described [59]. 5µl rabbit anti-histone H3K4me3 antibody (Millipore) was coupled to 20µl Dynabeads-Protein A (Life Technologies) for each ChIP. DNA was purified using MinElute PCR Purification Kits (Qiagen), eluted in 20µl buffer EB, and diluted 1:10 for quantitative PCR (qPCR) using SYBR Select Master Mix (Applied Biosystems). ChIP and input samples from three biological replicates of *Tex19*.*1*^*+/±*^ and *Tex19*.*1*^*-/-*^ P16 testes were assayed in triplicate by qPCR using SYBR Green Master Mix (Roche) and a LightCycler 480 (Roche). ChIP enrichment was calculated relative to 10% input samples, and normalised to enrichment for the β-actin (*Actb*) transcriptional start site. Primers used for qPCR are listed in S2 Table. Primers for *Prdm9*-dependent recombination hotspots were derived from DMC1 ChIP-seq data [5].

### Histology and in situ hybridisation

Histology of Bouin’s-fixed testes, and in situ hybridisation of MMERVK10C probes to Bouin’s-fixed testis sections were performed as described [24].

### qRT-PCR

RNA was isolated from macerated mouse testes using TRIzol (Invitrogen) and treated with Turbo DNAse (Ambion) to digest any genomic DNA contamination. 1µg DNAse-treated RNA was used to synthesise cDNA using Superscript III (Invitrogen). The cDNA was used as a template for qPCR using SYBR Select Master Mix (Applied Biosystems), and the relative quantity of RNA transcript calculated using the standard curve method as described by the supplier. The qPCR was performed on the LightCycler 480 (Roche), retrotransposon RNA levels were measured relative to β-actin, and normalised to control samples. Each biological replicate was assayed in triplicate, and alongside no reverse transcriptase and no template control reactions to confirm the absence of genomic DNA contamination.

### Western blotting

P16 testes were homogenised in 2× Laemmli SDS sample buffer (Sigma) with a motorised pestle, boiled for 2–5 minutes and insoluble material pelleted in a microcentrifuge. Lysates were resolved by electrophoresis through pre-cast Bis-Tris polyacrylamide gels (Life Technologies) and Western blotted to PVDF membrane using the iBlot Dry Blotting System (Life Technologies). PBS containing 5% skimmed milk and 0.1% Tween was used to block the membrane and dilute antibodies. Primary antibodies for Western blotting were mouse anti-UBR2 (Abcam, 1:1000 dilution) and mouse anti-β-actin (Sigma, 1:5000 dilution). HRP-conjugated secondary antibodies (Cell Signaling Technology; Bio-Rad) were detected with SuperSignal West Pico Chemiluminescent Substrate (Thermo Scientific).

## Supporting information

S1 Table Antibodies. Primary antibodies used for immunostaining meiotic chromosome spreads.
(PDF)

S2 Table Primer sequences. Sequences of oligonucleotide primers used for H3K4me3 ChIP, qRT-PCR, and CRISPR/Cas9-mediated generation and genotyping of *Ubr2*^-/-^ mice. Lower case nucleotides in the CRISPR/Cas9 repair template represent mutations introduced into the UBR domain of *Ubr2*.
(PDF)

S1 Fig Synapsis progression and accumulation of recombination foci on asynapsed chromatin in *Tex19*.*1*^*-/-*^ spermatocytes. (A) Chromosome spreads from *Tex19*.*1*^*+/±*^ and *Tex19*.*1*^*-/-*^ zygotene spermatocytes immunostained for synaptonemal complex (SC) components SYCP3 (red) and SYCE2 (green). The extent of synapsis was measured by assessing the amount of fully assembled SC marked by SYCP3 and SYCE2 relative to the amount of axial element containing SYCP3 only. Representative images of *Tex19*.*1*^*+/±*^ and *Tex19*.*1*^*-/-*^ nuclei with 10–30% and <10% synapsis respectively are shown. Scale bar 10 μm. (B) Classification of zygotene nuclei based on the extent of synapsis. SYCP3 and SYCE2 were used to visualise axial elements and assess the extent of synapsis respectively. Zygotene nuclei were distinguished from leptotene nuclei by complete axial element formation, and from asynapsed pachytene nuclei by the absence of any completely synapsed autosomes. Data represents scoring from 22 zygotene *Tex19*.*1*^*-/-*^ nuclei and 19 controls. (C) Chromosome spreads from *Tex19*.*1*^*+/±*^ and *Tex19*.*1*^*-/-*^ pachytene spermatocytes immunostained to visualise recombination foci on the sex chromosomes. The sex chromosomes are labelled with SYCP3 (red) but not SYCE2 (blue). RPA (green) was used to mark recombination foci. Scale bar 10 μm. (D) Beeswarm plots showing number of RPA foci associated with the sex chromosomes in fully synapsed pachytene *Tex19*.*1*^*+/±*^ and fully synapsed pachytene *Tex19*.*1*^*-/-*^ nuclei. The number of XY-associated RPA foci is not significantly different (6.8±0.4 and 7.0±0.4 foci respectively, n = 104,88 from three mice per genotype; Mann-Whitney U test).
(TIF)

S2 Fig *Spo11*-independent RAD51 foci do not accumulate on chromosome axes in the absence of *Tex19*.*1*. (A) Immunostaining of chromosome spreads from zygotene-like *Spo11*^*-/-*^ *Tex19*.*1*^*+/±*^ and *Spo11*^*-/-*^ *Tex19*.*1*^*-/-*^ spermatocytes for the SC component SYCP3 (red) to identify chromosome axes, and RAD51 (green) to mark recombination foci and sites of DNA damage. Scale bar 10 μm. (B) Quantification of the number of axis-associated RAD51 foci in zygotene-like *Spo11*^*-/-*^ *Tex19*.*1*^*+/±*^ and *Spo11*^*-/-*^ *Tex19*.*1*^*-/-*^ spermatocytes. n = 71, 73 from two *Spo11*^*-/-*^ *Tex19*.*1*^*+/±*^ and two *Spo11*^*-/-*^ *Tex19*.*1*^*-/-*^ animals. Means are indicated with horizontal bars. Control *Spo11*^*-/-*^ *Tex19*.*1*^*+/±*^ zygotene-like spermatocytes have 1.4±1.7 axis-associated RAD51 foci, *Spo11*^*-/-*^ *Tex19*.*1*^*-/-*^ zygotene-like spermatocytes have 1.1±2.2 axis-associated RAD51 foci. Some non-axis associated RAD51 foci are present in these nuclei, which could potentially represent background staining with this antibody and as the number of axis-associated RAD51 foci in these nuclei is very low, we cannot exclude the possibility that some axis-associated RAD51 foci counted in these data represent background staining. In addition, the large proportion (64%) of nuclei containing no RAD51 foci in the data precludes meaningful analysis by Mann-Whitney U test. However, in contrast to *Mael*^*-/-*^ *Spo11*^*-/-*^ spermatocytes [16], *Tex19*.*1*^*-/-*^ *Spo11*^*-/-*^ spermatocytes do not accumulate large numbers of RAD51 foci on their axes.
(TIF)

S3 Fig Loss of *Tex19*.*1* does not detectably affect early recombination foci in female meiosis. (A) Immunostaining of chromosome spreads from E14.5 *Tex19*.*1*^*+/±*^ and *Tex19*.*1*^*-/-*^ foetal oocytes for the SC component SYCP3 (red) to identify late leptotene nuclei and fragments of chromosome axes, and RAD51 (green) to mark recombination foci. Scale bar 10 μm. (B) Quantification of the number of RAD51-positive recombination foci in late leptotene *Tex19*.*1*^*+/±*^ and *Tex19*.*1*^*-/-*^ oocytes. n = 24, 15 from four *Tex19*.*1*^*+/±*^ and three *Tex19*.*1*^*-/-*^ foetuses. Means are indicated with horizontal bars, and ns indicates no significant difference (Mann-Whitney U test). Control *Tex19*.*1*^*+/±*^ late leptotene nuclei have 100±16 RAD51 foci, *Tex19*.*1*^*-/-*^ leptotene nuclei have 115±14 RAD51 foci.
(TIF)

S4 Fig Spermatogenesis defects in *Ubr2*^*-/-*^ mice. (A) Western blot for UBR2 in P16 *Ubr2*^*-/-*^ testes. *Ubr2*^*-/-*^ testes have no detectable UBR2 protein. β-actin is shown as a loading control. Migration of molecular weight markers (kDa) is shown on the left of the blots. (B, C) Testis weight and epididymal sperm counts are reduced in *Ubr2*^*-/-*^ mice. Testis weight is 74.5±1.3 mg in control but 24.1±0.9 mg in *Ubr2*^*-/-*^ mice (p<0.05, n = 6,6; Student's t-test). Sperm count is 1.0±0.3 × 10^7^ sperm per epididymis in control mice but undetectable in *Ubr2*^*-/-*^ mice (p<0.05, n = 3, 3; Student's t-test). (D) Testis histology in *Ubr2*^*-/-*^ mice. Defects in spermatogenesis are apparent in haematoxylin and eosin-stained sections of *Ubr2*^*-/-*^ testes. *Ubr2*^*-/-*^ testis tubules contain reduced numbers of round spermatids (arrows) and elongated spermatids (arrowheads) relative to controls, although these spermatogenic stages are not completely absent. *Ubr2*^*-/-*^ tubules also exhibit pyknotic nuclei (asterisks) and an accumulation of zygotene-like cells (Z) indicative of defects in progression through meiotic prophase. Scale bar 100 μm. (E) Chromosome spreads from *Ubr2*^*+/+*^ and *Ubr2*^*-/-*^ zygotene spermatocytes immunostained for synaptonemal complex (SC) components SYCP3 (red) and SYCP1 (green). The extent of synapsis was measured by assessing the amount of fully assembled SC marked by SYCP3 and SYCP1 relative to the amount of axial element containing SYCP3 only. Representative images of *Ubr2*^*+/+*^ and *Ubr2*^*-/-*^ nuclei with 10–30% and <10% synapsis respectively are shown. Scale bar 10 μm. (F) Classification of zygotene nuclei based on the extent of synapsis. SYCP3 and SYCP1 were used to visualise axial elements and assess the extent of synapsis respectively. Zygotene nuclei were distinguished from leptotene nuclei by the presence of stretches of synapsis, and from asynapsed pachytene nuclei by the absence of any completely synapsed autosomes. Data represents scoring from 56 *Ubr2*^*+/+*^ and 75 *Ubr2*^*-/-*^ zygotene nuclei across three mice per genotype.
(TIF)
