# Supplementary material for: Tex19.1 promotes Spo11-dependent meiotic recombination in mouse spermatocytes
Source: PLoS Genet. 2017 Jul 14;13(7):e1006904. doi: 10.1371/journal.pgen.1006904 (PMC5533463; doi:10.1371/journal.pgen.1006904)
Supplement: S1 Table — Primary antibodies used for immunostaining meiotic chromosome spreads. (PDF) [file pgen.1006904.s001.pdf]

Antibody	Species	Source	Dilution
Anti-DMC1	Rabbit	Santa Cruz, H-100, sc-22768	1:50
Anti-MEI4	Rabbit	Bernard de Massy, IGH, Montpellier, France	1:200
Anti-RPA	Rabbit	C. James Ingles, University of Toronto, Toronto, Canada	1:300
Anti-SYCE2	Guinea Pig	Howard Cooke, MRC HGU, Edinburgh, UK	1:1000
Anti-SYCP1	Guinea Pig	Howard Cooke, MRC HGU, Edinburgh, UK	1:200
Anti-SYCP1	Rabbit	Abcam, ab15090	1:200
Anti-SYCP3	Mouse	Santa Cruz, D-1, sc-74569	1:200
Anti-SYCP3	Mouse	Abcam, ab97672	1:500
Anti-SYCP3	Rabbit	Abcam, ab1592	1:300
Anti-SYCP3	Rabbit	LS Bio, LS-B175	1:500
Anti-γH2AX	Mouse	Millipore, JBW301	1:3000
Anti-γH2AX	Rabbit	Millipore, 07-164	1:200
Anti-H3K4me3	Rabbit	Millipore, 04-745	1:100
Anti-RAD51	Rabbit	Calbiochem, PC130	1:500
