# Supplementary material for: Tex19.1 promotes Spo11-dependent meiotic recombination in mouse spermatocytes
Source: PLoS Genet. 2017 Jul 14;13(7):e1006904. doi: 10.1371/journal.pgen.1006904 (PMC5533463; doi:10.1371/journal.pgen.1006904)
Supplement: S2 Table — Sequences of oligonucleotide primers used for H3K4me3 ChIP, qRT-PCR, and CRISPR/Cas9-mediated generation and genotyping of Ubr2-/- mice. Lower case nucleotides in the CRISPR/Cas9 repair template represent mutations introduced into the UBR domain of Ubr2. (PDF) [file pgen.1006904.s002.pdf]

Target	Forward Primer	Reverse Primer
Actb TSS ChIP	CCTCGATGCTGACCCTCATCC	GACACTGCCCCATTCAATGTCTC
Gapdh TSS ChIP	TCCCTAGACCCGTACAGTGC	CTCTGCTCCTCCCTGTTCC
Polr2a TSS ChIP	GACTCCGAACTGCACTCTCT	CAGCCTTTCCCTCCCTATCC
Polr2a Intragenic ChIP	CTGTCTCCAGGTCATTGAGAAGG	GTTGATCTTGGAACCCTTGGCTCC
Hotspot 10qC2 ChIP	GCCAAGTAAGCTCTTCCTCC	TGACTTCTGCCTACCACTCA
Hotspot 12qA2 ChIP	CCACCCCAAACTTTTCCGTA	TGGCCCTTCTGTCTTTGAAC
Hotspot 12qA1.1 ChIP	AACGTCCAGCCTAATTGTCC	GAATCAGGACCTCAGGCAAG
MMERVK10C LTR ChIP	CTGAGTGGCACTGACTACTG	AGCCTCATTTGCATGTTCCT
IAP LTR ChIP	GATGGTGCTGACATCCTGTG	CTGACGTTCACGGGAAAAAC
LINE-1 5'UTR ChIP	AATCTGTCTCCCAGGTCTGC	CCTTTCGCCATCTGGTAATC
Actb qRT-PCR	GGCTGTATTCCCCTCCATCG	ACATGGCATTGTTACCAACTGG
MMERVK10C qRT-PCR	AACTGGTCGCAGGAGCTG	GGTAAAGTCTCCGAGGGTCA
IAP qRT-PCR	GCACCCTCAAAGCCTATCTTA	TCCCTTGGTCAGTCTGGATTT
LINE-1 qRT-PCR	GGAGGGACATTTCATTCTCATC	GCTGCTCTTGTATTTGGAGCATAGA
Ubr2 genotyping	TCTGAGGTTGCAAGAGAATGT	GGCCACAGATCAGCTAAACC
Ubr2 CRISPR guide A	AAACGTGTTTTATGCATGGAGTGCC	CACCGGCACTCCATGCATAAAACAC
Ubr2 CRISPR guide B	AAACATATCGATGGTCTCTATGGAC	CACCGTCCATAGAGACCATCGATAT
CRISPR guide A T7 PCR	TGTAATACGACTCACTATAGGGAAACGTG TTTTATGCATGGAGTGCC	AAAAGCACCGACTCGGTGCC
CRISPR guide B T7 PCR	TGTAATACGACTCACTATAGGGAAACATA TCGATGGTCTCTATGGAC	AAAAGCACCGACTCGGTGCC
CRISPR repair template	GACTGTGCAGTTGACCCCAtCTagaTTTTATGCATGGAGTGCTTCCTGGGAAGTATCCATA GAGACCATCGATATAGG	
