# Supplementary figures and images for: Tex19.1 promotes Spo11-dependent meiotic recombination in mouse spermatocytes
Source: PLoS Genet. 2017 Jul 14;13(7):e1006904. doi: 10.1371/journal.pgen.1006904 (PMC5533463; doi:10.1371/journal.pgen.1006904)

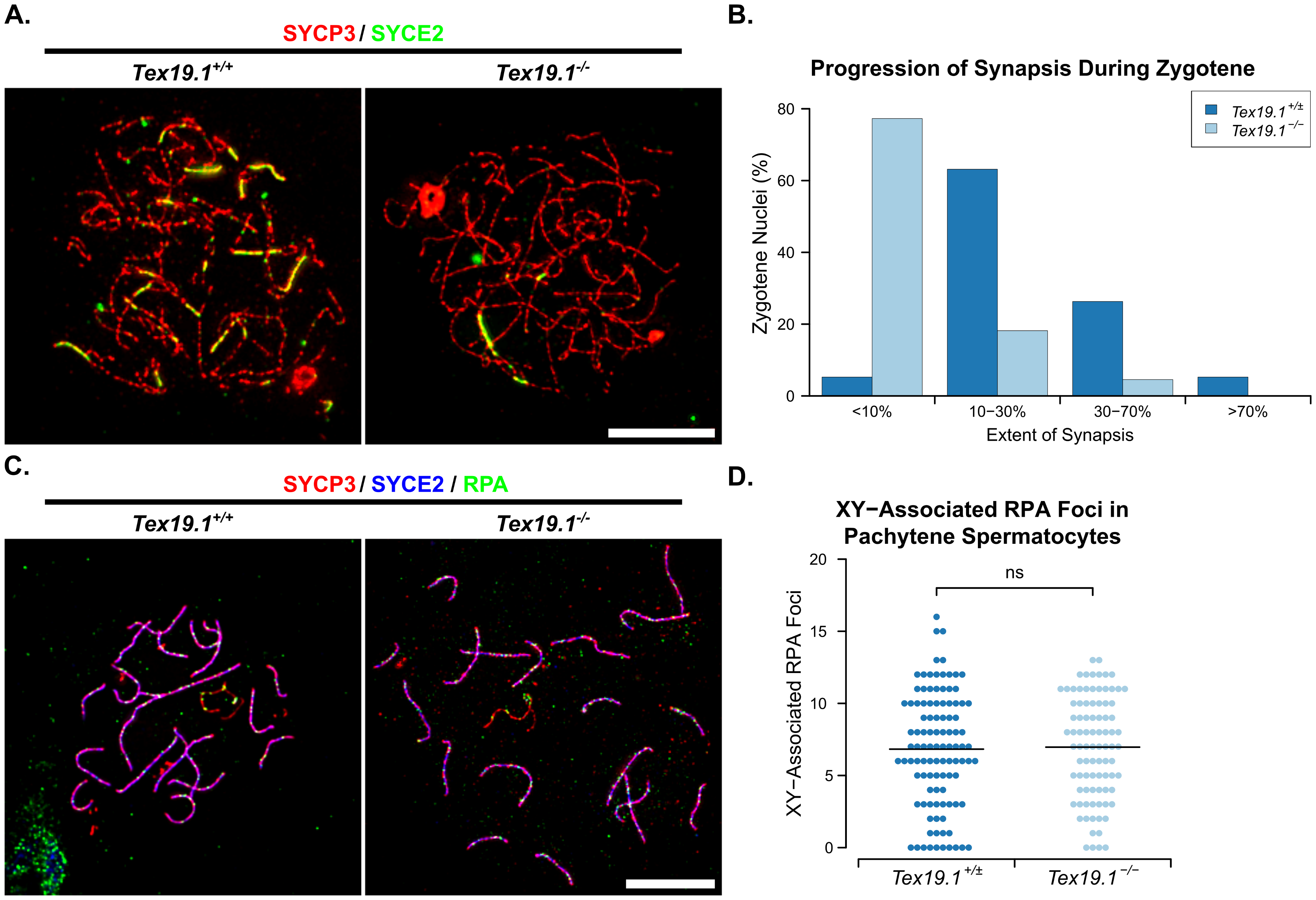

Supplement: S1 Fig — (A) Chromosome spreads from Tex19.1+/± and Tex19.1-/- zygotene spermatocytes immunostained for synaptonemal complex (SC) components SYCP3 (red) and SYCE2 (green). The extent of synapsis was measured by assessing the amount of fully assembled SC marked by SYCP3 and SYCE2 relative to the amount of axial element containing SYCP3 only. Representative images of Tex19.1+/± and Tex19.1-/- nuclei with 10–30% and <10% synapsis respectively are shown. Scale bar 10 μm. (B) Classification of zygotene nuclei based on the extent of synapsis. SYCP3 and SYCE2 were used to visualise axial elements and assess the extent of synapsis respectively. Zygotene nuclei were distinguished from leptotene nuclei by complete axial element formation, and from asynapsed pachytene nuclei by the absence of any completely synapsed autosomes. Data represents scoring from 22 zygotene Tex19.1-/- nuclei and 19 controls. (C) Chromosome spreads from Tex19.1+/± and Tex19.1-/- pachytene spermatocytes immunostained to visualise recombination foci on the sex chromosomes. The sex chromosomes are labelled with SYCP3 (red) but not SYCE2 (blue). RPA (green) was used to mark recombination foci. Scale bar 10 μm. (D) Beeswarm plots showing number of RPA foci associated with the sex chromosomes in fully synapsed pachytene Tex19.1+/± and fully synapsed pachytene Tex19.1-/- nuclei. The number of XY-associated RPA foci is not significantly different (6.8±0.4 and 7.0±0.4 foci respectively, n = 104,88 from three mice per genotype; Mann-Whitney U test). (TIF) [file pgen.1006904.s003.tif]

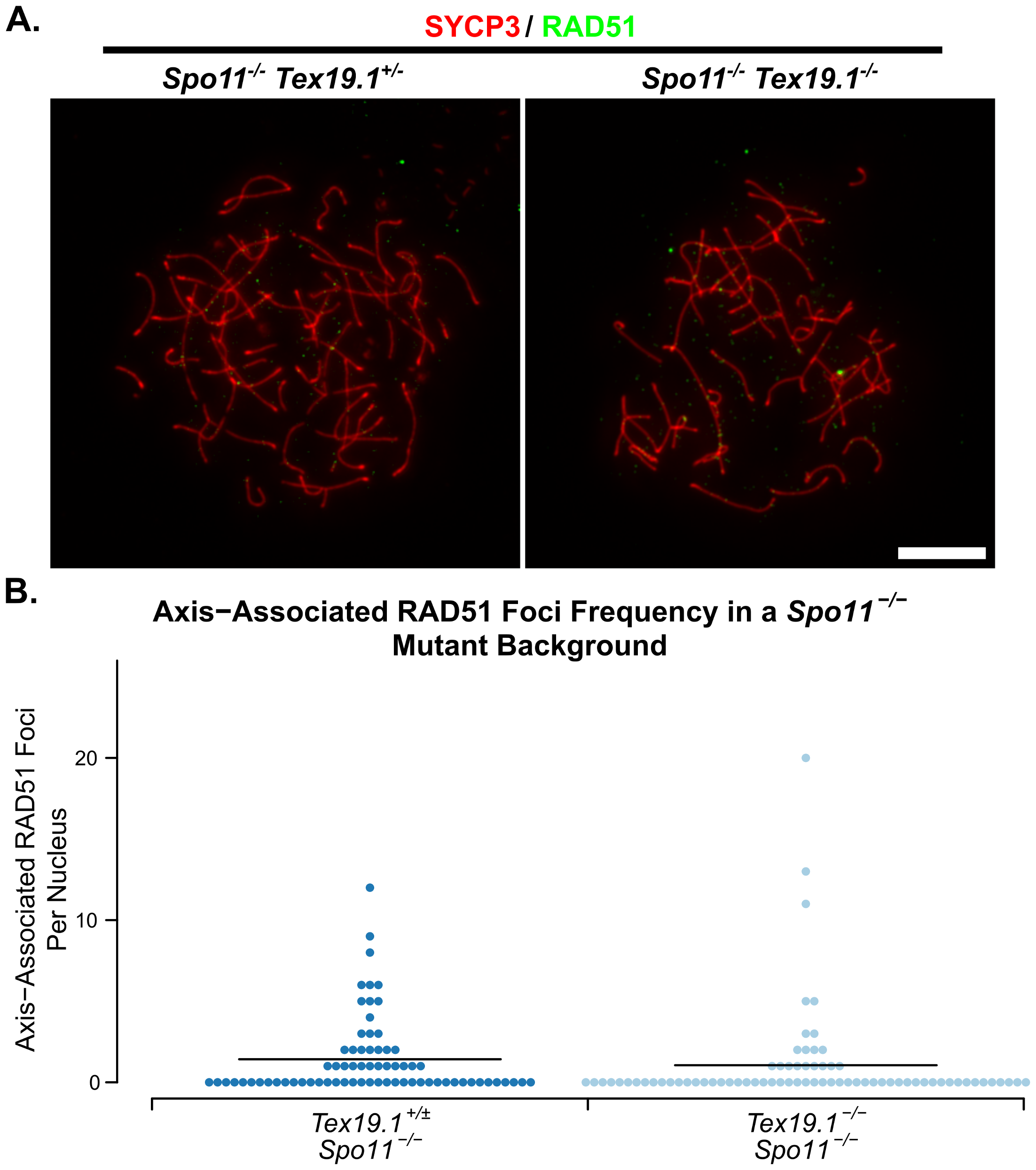

Supplement: S2 Fig — (A) Immunostaining of chromosome spreads from zygotene-like Spo11-/- Tex19.1+/± and Spo11-/- Tex19.1-/- spermatocytes for the SC component SYCP3 (red) to identify chromosome axes, and RAD51 (green) to mark recombination foci and sites of DNA damage. Scale bar 10 μm. (B) Quantification of the number of axis-associated RAD51 foci in zygotene-like Spo11-/- Tex19.1+/± and Spo11-/- Tex19.1-/- spermatocytes. n = 71, 73 from two Spo11-/- Tex19.1+/± and two Spo11-/- Tex19.1-/- animals. Means are indicated with horizontal bars. Control Spo11-/- Tex19.1+/± zygotene-like spermatocytes have 1.4±1.7 axis-associated RAD51 foci, Spo11-/- Tex19.1-/- zygotene-like spermatocytes have 1.1±2.2 axis-associated RAD51 foci. Some non-axis associated RAD51 foci are present in these nuclei, which could potentially represent background staining with this antibody and as the number of axis-associated RAD51 foci in these nuclei is very low, we cannot exclude the possibility that some axis-associated RAD51 foci counted in these data represent background staining. In addition, the large proportion (64%) of nuclei containing no RAD51 foci in the data precludes meaningful analysis by Mann-Whitney U test. However, in contrast to Mael-/- Spo11-/- spermatocytes [16], Tex19.1-/- Spo11-/- spermatocytes do not accumulate large numbers of RAD51 foci on their axes. (TIF) [file pgen.1006904.s004.tif]

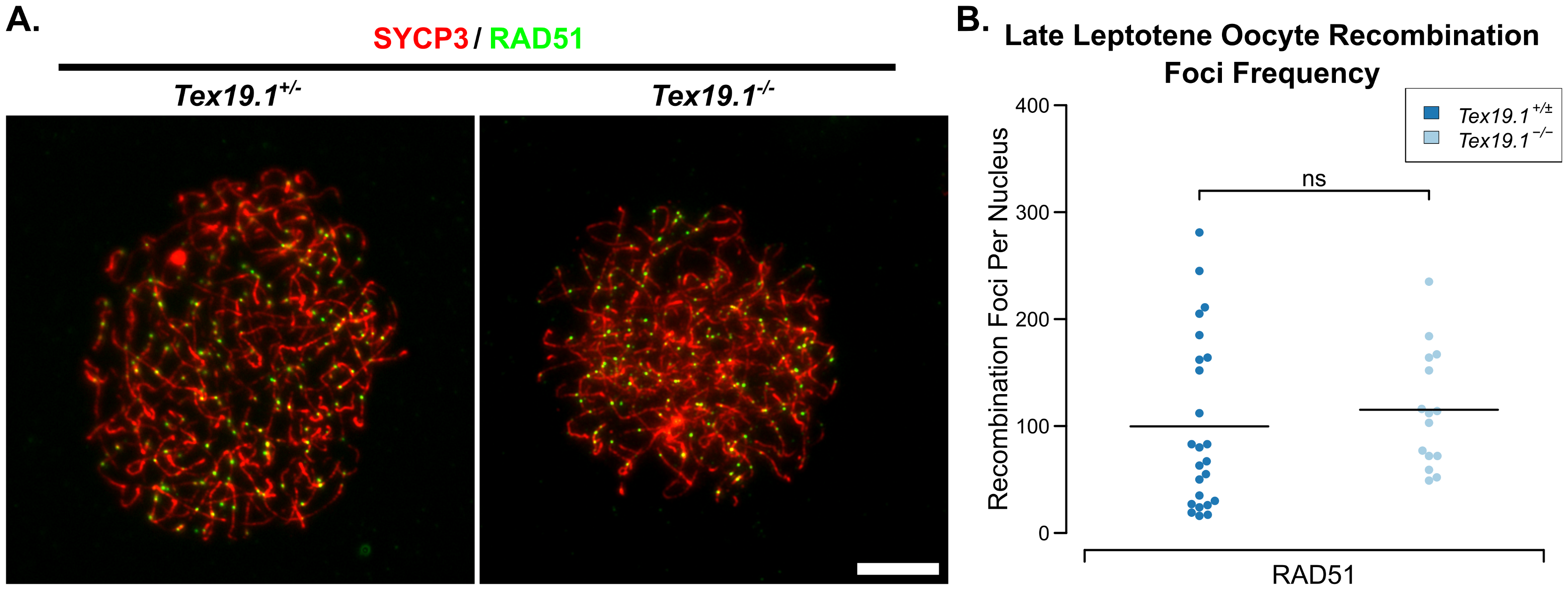

Supplement: S3 Fig — (A) Immunostaining of chromosome spreads from E14.5 Tex19.1+/± and Tex19.1-/- foetal oocytes for the SC component SYCP3 (red) to identify late leptotene nuclei and fragments of chromosome axes, and RAD51 (green) to mark recombination foci. Scale bar 10 μm. (B) Quantification of the number of RAD51-positive recombination foci in late leptotene Tex19.1+/± and Tex19.1-/- oocytes. n = 24, 15 from four Tex19.1+/± and three Tex19.1-/- foetuses. Means are indicated with horizontal bars, and ns indicates no significant difference (Mann-Whitney U test). Control Tex19.1+/± late leptotene nuclei have 100±16 RAD51 foci, Tex19.1-/- leptotene nuclei have 115±14 RAD51 foci. (TIF) [file pgen.1006904.s005.tif]

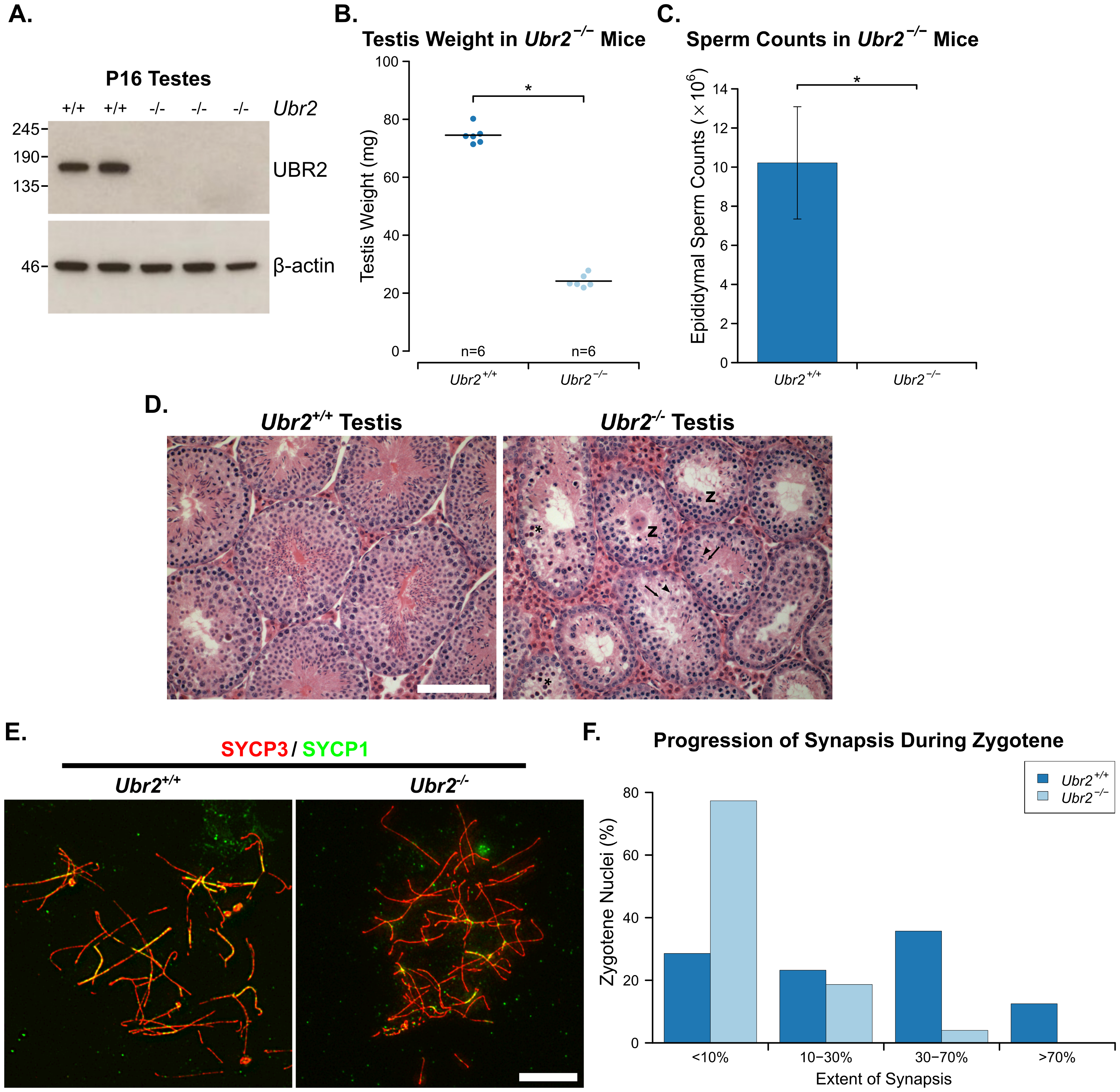

Supplement: S4 Fig — (A) Western blot for UBR2 in P16 Ubr2-/- testes. Ubr2-/- testes have no detectable UBR2 protein. β-actin is shown as a loading control. Migration of molecular weight markers (kDa) is shown on the left of the blots. (B, C) Testis weight and epididymal sperm counts are reduced in Ubr2-/- mice. Testis weight is 74.5±1.3 mg in control but 24.1±0.9 mg in Ubr2-/- mice (p<0.05, n = 6,6; Student's t-test). Sperm count is 1.0±0.3 × 10^7^ sperm per epididymis in control mice but undetectable in Ubr2-/- mice (p<0.05, n = 3, 3; Student's t-test). (D) Testis histology in Ubr2-/- mice. Defects in spermatogenesis are apparent in haematoxylin and eosin-stained sections of Ubr2-/- testes. Ubr2-/- testis tubules contain reduced numbers of round spermatids (arrows) and elongated spermatids (arrowheads) relative to controls, although these spermatogenic stages are not completely absent. Ubr2-/- tubules also exhibit pyknotic nuclei (asterisks) and an accumulation of zygotene-like cells (Z) indicative of defects in progression through meiotic prophase. Scale bar 100 μm. (E) Chromosome spreads from Ubr2+/+ and Ubr2-/- zygotene spermatocytes immunostained for synaptonemal complex (SC) components SYCP3 (red) and SYCP1 (green). The extent of synapsis was measured by assessing the amount of fully assembled SC marked by SYCP3 and SYCP1 relative to the amount of axial element containing SYCP3 only. Representative images of Ubr2+/+ and Ubr2-/- nuclei with 10–30% and <10% synapsis respectively are shown. Scale bar 10 μm. (F) Classification of zygotene nuclei based on the extent of synapsis. SYCP3 and SYCP1 were used to visualise axial elements and assess the extent of synapsis respectively. Zygotene nuclei were distinguished from leptotene nuclei by the presence of stretches of synapsis, and from asynapsed pachytene nuclei by the absence of any completely synapsed autosomes. Data represents scoring from 56 Ubr2+/+ and 75 Ubr2-/- zygotene nuclei across three mice per genotype. (TIF) [file pgen.1006904.s006.tif]
